# Atrial fibrillation-associated electrical remodelling in human induced pluripotent stem cell-derived atrial cardiomyocytes: a novel pathway for antiarrhythmic therapy development

**DOI:** 10.1093/cvr/cvad143

**Published:** 2023-09-07

**Authors:** Fitzwilliam Seibertz, Tony Rubio, Robin Springer, Fiona Popp, Melanie Ritter, Aiste Liutkute, Lena Bartelt, Lea Stelzer, Fereshteh Haghighi, Jan Pietras, Hendrik Windel, Núria Díaz i Pedrosa, Markus Rapedius, Yannic Doering, Richard Solano, Robin Hindmarsh, Runzhu Shi, Malte Tiburcy, Tobias Bruegmann, Ingo Kutschka, Katrin Streckfuss-Bömeke, George Kensah, Lukas Cyganek, Wolfram H Zimmermann, Niels Voigt

**Affiliations:** Institute of Pharmacology and Toxicology, University Medical Center Göttingen, Georg-August University Göttingen, Robert-Koch-Straße 40, 37075 Göttingen, Germany; DZHK (German Center for Cardiovascular Research), partner site Göttingen, Germany; Cluster of Excellence ‘Multiscale Bioimaging: from Molecular Machines to Networks of Excitable Cells’ (MBExC), University of Göttingen, Göttingen, Germany; Institute of Pharmacology and Toxicology, University Medical Center Göttingen, Georg-August University Göttingen, Robert-Koch-Straße 40, 37075 Göttingen, Germany; DZHK (German Center for Cardiovascular Research), partner site Göttingen, Germany; Institute of Pharmacology and Toxicology, University Medical Center Göttingen, Georg-August University Göttingen, Robert-Koch-Straße 40, 37075 Göttingen, Germany; DZHK (German Center for Cardiovascular Research), partner site Göttingen, Germany; Institute of Pharmacology and Toxicology, University Medical Center Göttingen, Georg-August University Göttingen, Robert-Koch-Straße 40, 37075 Göttingen, Germany; DZHK (German Center for Cardiovascular Research), partner site Göttingen, Germany; Institute of Pharmacology and Toxicology, University Medical Center Göttingen, Georg-August University Göttingen, Robert-Koch-Straße 40, 37075 Göttingen, Germany; DZHK (German Center for Cardiovascular Research), partner site Göttingen, Germany; Institute of Pharmacology and Toxicology, University Medical Center Göttingen, Georg-August University Göttingen, Robert-Koch-Straße 40, 37075 Göttingen, Germany; DZHK (German Center for Cardiovascular Research), partner site Göttingen, Germany; Institute of Pharmacology and Toxicology, University Medical Center Göttingen, Georg-August University Göttingen, Robert-Koch-Straße 40, 37075 Göttingen, Germany; DZHK (German Center for Cardiovascular Research), partner site Göttingen, Germany; Institute of Pharmacology and Toxicology, University Medical Center Göttingen, Georg-August University Göttingen, Robert-Koch-Straße 40, 37075 Göttingen, Germany; DZHK (German Center for Cardiovascular Research), partner site Göttingen, Germany; DZHK (German Center for Cardiovascular Research), partner site Göttingen, Germany; Department of Cardiothoracic and Vascular Surgery, Georg-August-University Göttingen, Göttingen, Germany; DZHK (German Center for Cardiovascular Research), partner site Göttingen, Germany; Department of Cardiothoracic and Vascular Surgery, Georg-August-University Göttingen, Göttingen, Germany; DZHK (German Center for Cardiovascular Research), partner site Göttingen, Germany; Department of Cardiothoracic and Vascular Surgery, Georg-August-University Göttingen, Göttingen, Germany; Institute of Pharmacology and Toxicology, University Medical Center Göttingen, Georg-August University Göttingen, Robert-Koch-Straße 40, 37075 Göttingen, Germany; DZHK (German Center for Cardiovascular Research), partner site Göttingen, Germany; Nanion Technologies GmbH, Munich, Germany; Institute of Pharmacology and Toxicology, University Medical Center Göttingen, Georg-August University Göttingen, Robert-Koch-Straße 40, 37075 Göttingen, Germany; DZHK (German Center for Cardiovascular Research), partner site Göttingen, Germany; Institute of Pharmacology and Toxicology, University Medical Center Göttingen, Georg-August University Göttingen, Robert-Koch-Straße 40, 37075 Göttingen, Germany; DZHK (German Center for Cardiovascular Research), partner site Göttingen, Germany; Department of Cardiothoracic and Vascular Surgery, Georg-August-University Göttingen, Göttingen, Germany; DZHK (German Center for Cardiovascular Research), partner site Göttingen, Germany; Clinic for Cardiology and Pneumology, University Medical Center Göttingen, Georg-August University Göttingen, Germany; DZHK (German Center for Cardiovascular Research), partner site Göttingen, Germany; Institute for Cardiovascular Physiology, University Medical Center Göttingen, Göttingen, Germany; Institute of Pharmacology and Toxicology, University Medical Center Göttingen, Georg-August University Göttingen, Robert-Koch-Straße 40, 37075 Göttingen, Germany; DZHK (German Center for Cardiovascular Research), partner site Göttingen, Germany; DZHK (German Center for Cardiovascular Research), partner site Göttingen, Germany; Cluster of Excellence ‘Multiscale Bioimaging: from Molecular Machines to Networks of Excitable Cells’ (MBExC), University of Göttingen, Göttingen, Germany; Institute for Cardiovascular Physiology, University Medical Center Göttingen, Göttingen, Germany; DZHK (German Center for Cardiovascular Research), partner site Göttingen, Germany; Department of Cardiothoracic and Vascular Surgery, Georg-August-University Göttingen, Göttingen, Germany; DZHK (German Center for Cardiovascular Research), partner site Göttingen, Germany; Clinic for Cardiology and Pneumology, University Medical Center Göttingen, Georg-August University Göttingen, Germany; Institute of Pharmacology and Toxicology, University of Würzburg, Würzburg, Germany; DZHK (German Center for Cardiovascular Research), partner site Göttingen, Germany; Department of Cardiothoracic and Vascular Surgery, Georg-August-University Göttingen, Göttingen, Germany; DZHK (German Center for Cardiovascular Research), partner site Göttingen, Germany; Cluster of Excellence ‘Multiscale Bioimaging: from Molecular Machines to Networks of Excitable Cells’ (MBExC), University of Göttingen, Göttingen, Germany; Clinic for Cardiology and Pneumology, University Medical Center Göttingen, Georg-August University Göttingen, Germany; Institute of Pharmacology and Toxicology, University Medical Center Göttingen, Georg-August University Göttingen, Robert-Koch-Straße 40, 37075 Göttingen, Germany; DZHK (German Center for Cardiovascular Research), partner site Göttingen, Germany; Cluster of Excellence ‘Multiscale Bioimaging: from Molecular Machines to Networks of Excitable Cells’ (MBExC), University of Göttingen, Göttingen, Germany; German Center for Neurodegenerative Diseases (DZNE), Göttingen, Germany; Fraunhofer Institute for Translational Medicine and Pharmacology (ITMP), Göttingen, Germany; Campus-Institute Data Science (CIDAS), University of Göttingen, Göttingen, Germany; Institute of Pharmacology and Toxicology, University Medical Center Göttingen, Georg-August University Göttingen, Robert-Koch-Straße 40, 37075 Göttingen, Germany; DZHK (German Center for Cardiovascular Research), partner site Göttingen, Germany; Cluster of Excellence ‘Multiscale Bioimaging: from Molecular Machines to Networks of Excitable Cells’ (MBExC), University of Göttingen, Göttingen, Germany

**Keywords:** Stem cells, Ion channel, Action potential, Atrial fibrillation, Optogenetics

## Abstract

**Aims:**

Atrial fibrillation (AF) is associated with tachycardia-induced cellular electrophysiology alterations which promote AF chronification and treatment resistance. Development of novel antiarrhythmic therapies is hampered by the absence of scalable experimental human models that reflect AF-associated electrical remodelling. Therefore, we aimed to assess if AF-associated remodelling of cellular electrophysiology can be simulated in human atrial-like cardiomyocytes derived from induced pluripotent stem cells in the presence of retinoic acid (iPSC-aCM), and atrial-engineered human myocardium (aEHM) under short term (24 h) and chronic (7 days) tachypacing (TP).

**Methods and results:**

First, 24-h electrical pacing at 3 Hz was used to investigate whether AF-associated remodelling in iPSC-aCM and aEHM would ensue. Compared to controls (24 h, 1 Hz pacing) TP-stimulated iPSC-aCM presented classical hallmarks of AF-associated remodelling: (i) decreased L-type Ca^2+^ current (I_Ca,L_) and (ii) impaired activation of acetylcholine-activated inward-rectifier K^+^ current (I_K,ACh_). This resulted in action potential shortening and an absent response to the M-receptor agonist carbachol in both iPSC-aCM and aEHM subjected to TP. Accordingly, mRNA expression of the channel-subunit Kir3.4 was reduced. Selective I_K,ACh_ blockade with tertiapin reduced basal inward-rectifier K^+^ current only in iPSC-aCM subjected to TP, thereby unmasking an agonist-independent constitutively active I_K,ACh_. To allow for long-term TP, we developed iPSC-aCM and aEHM expressing the light-gated ion-channel f-Chrimson. The same hallmarks of AF-associated remodelling were observed after optical-TP. In addition, continuous TP (7 days) led to (i) increased amplitude of inward-rectifier K^+^ current (I_K1_), (ii) hyperpolarization of the resting membrane potential, (iii) increased action potential-amplitude and upstroke velocity as well as (iv) reversibly impaired contractile function in aEHM.

**Conclusions:**

Classical hallmarks of AF-associated remodelling were mimicked through TP of iPSC-aCM and aEHM. The use of the ultrafast f-Chrimson depolarizing ion channel allowed us to model the time-dependence of AF-associated remodelling *in vitro* for the first time. The observation of electrical remodelling with associated reversible contractile dysfunction offers a novel platform for human-centric discovery of antiarrhythmic therapies.

## Graphical Abstract

**Graphical Abstract:**
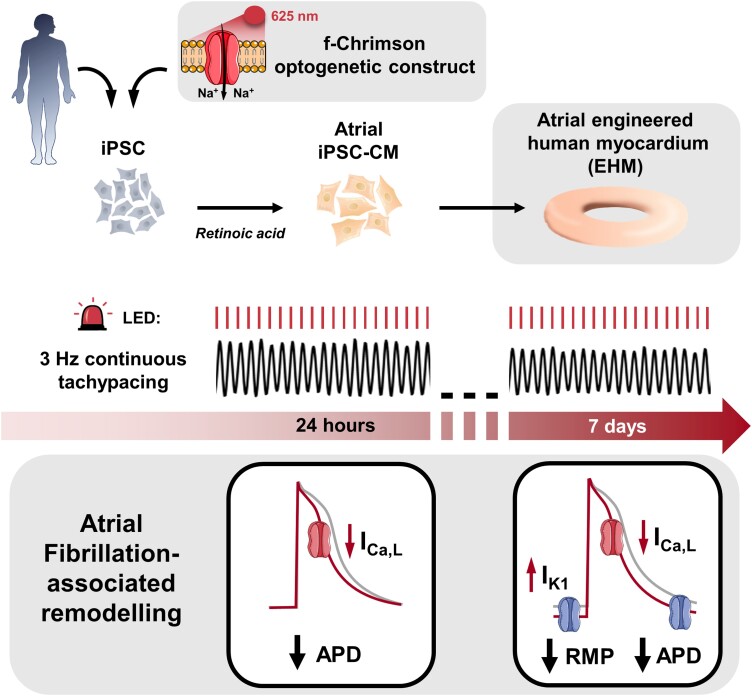

**Time of primary review: Days for primary review: days**

## 1. Introduction

Atrial fibrillation (AF) is the most frequently diagnosed cardiac arrhythmia and is associated with increased morbidity and mortality.^1^ Therapeutic interventions have major limitations, including limited efficacy and risk of life-threatening ventricular proarrhythmic side effects. Over the last decades, major insights into underlying molecular abnormalities contributing to the initiation, maintenance, and progression of AF have been gained.^1^ AF is associated with specific electrophysiological abnormalities that promote its sustainability and that are commonly summarized as electrical remodelling.^2^ Accordingly, action potential (AP) shortening is a major hallmark of AF-associated electrical remodelling.^3–5^

To date, many mechanistic studies of AF have mainly been based on experiments in animal models.^6,7^ However, the limitations of animal models are also apparent, including differences in basic cellular electrophysiology between humans and animals as well as the complexities underlying AF in patients. These limitations obviously limit the transferability of therapeutic interventions from animal to human.^8,9^ Alternatively, human atrial myocardium is available for fundamental studies of disease mechanisms, but cannot be maintained well *ex vivo* to study chronic triggers of AF or therapies. The development of atrial cardiomyocytes derived from human induced pluripotent stem cells (iPSC-aCM) promises to provide an unlimited source for *in vitro* studies of the molecular mechanisms of AF and therapeutic interventions.^10–12^ Similar to differences observed in native cardiomyocytes, iPSC-aCM show a distinct electrophysiological phenotype when compared to ventricular iPSC-CM (iPSC-vCM).^11,13,14^ Major differences include shorter AP duration (APD) and response to the vagal neurotransmitter acetylcholine, which is absent in ventricular cardiomyocytes.^11,14^ However, whether AF-associated electrical remodelling can be induced in iPSC-aCM is currently unknown.

iPSC-aCM in general and atrial-engineered human myocardium (aEHM) in particular represent novel promising tools for the development of new antiarrhythmic drugs and drug testing.^15–17^ Yet, without modelling the high frequencies of tachyarrhythmia and associated changes in ion channel function,^18,19^ their use in the development of novel antiarrhythmic therapies is limited. Importantly, AF-associated electrical remodelling has been shown to occur in a time-dependent manner, suggesting that chronic tachypacing (TP) would have to be introduced to simulate AF *in vitro*. For example, AF-associated increase in I_K1_ has been demonstrated to occur after 1 week or more in atrial TP^20^ whereas remodelling of L-type Ca^2+^ current (I_Ca,L_) appears to be an earlier event observed over days rather than weeks.^21^ iPSC-CM can be maintained and optically paced for longer cultivation periods^22^ and therefore may represent an ideal model to investigate long-term responses to atrial TP *in vitro*. We, therefore, tested the hypothesis that electrical remodelling can be induced in iPSC-aCM and aEHM by using electrical and optical TP. EHM resemble a macroscale tissue format with advanced organotypic maturation and contractile function.^11,23^

Here we show for the first time that major characteristics of AF-associated electrical remodelling including AP shorting, reduction of I_Ca,L_ and reduced vagal neurotransmitter response can be induced in iPSC-aCM subjected to 24 h TP.^24,25^ Furthermore, modelling chronic, uninterrupted TP (for 7 days), by making use of the newly developed ultrafast channelrhodopsin variant f-Chrimson,^26^ was also associated with additional upregulation of the basal inward-rectifier potassium current I_K1_ and consecutive hyperpolarization of the resting membrane potential (RMP), suggesting that AF-associated electrical remodelling is time-dependent and may therefore contribute to the progression of the arrhythmia and arrhythmia-associated contractile dysfunction. Taken together, our data suggest that increased atrial stimulation frequency is a major contributor to electrical remodelling observed in patients with AF and that underlying mechanisms can be modelled in iPSC-aCM and aEHM. Our novel human 2D and 3D models coupled with TP may therefore represent powerful *in vitro* tools to simulate AF, allowing for investigation of the mechanisms underlying AF pathophysiology and potentially phenotypic screens for novel antiarrhythmic therapies.

## 2. Methods

More detailed methods are provided in the Supplementary material*online*.

### 2.1 Human iPSC and differentiation into cardiomyocytes

**Figure 1:**
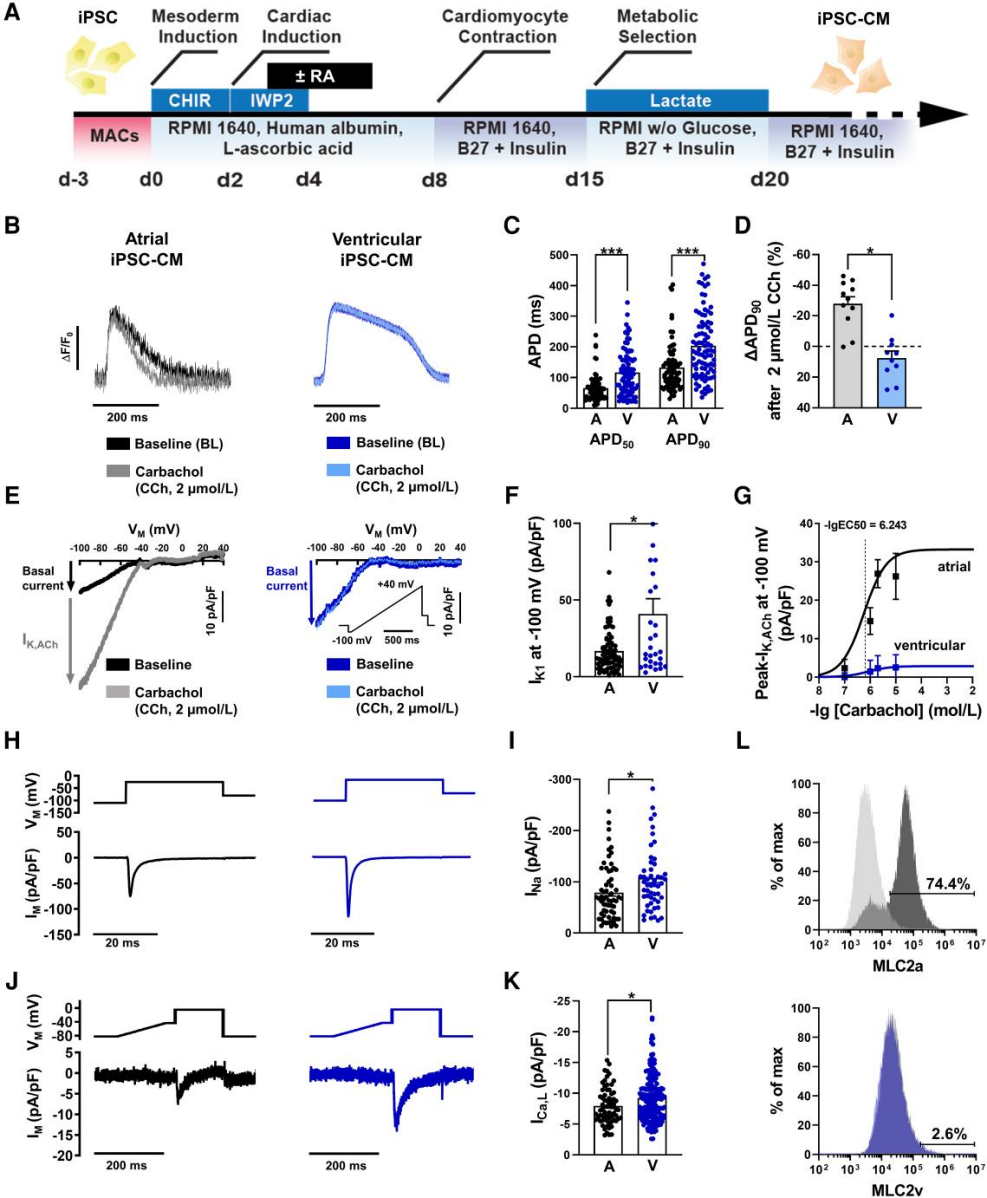
Cellular electrophysiology of atrial (iPSC-aCM) and ventricular (iPSC-vCM) induced pluripotent stem cell-derived cardiomyocytes. (*A*) Schematic of the iPSC-CM differentiation protocols used in this study. Application of 1 µmol/L retinoic acid (RA) from day 3 (d3) to day 6 induces an atrial specific subtype. (*B*) Representative optical action potentials (AP) elicited at 1 Hz in intact single iPSC-aCM (*A*, left) and iPSC-vCM (*V*, right) before (baseline) and after application of the M-receptor agonist carbachol (CCh, 2 µmol/L). (*C*) AP duration at 50 and 90% repolarization (APD_50_, APD_90_; atrial: *n* = 74/4, ventricular: *n* = 89/3). (*D*) Percentage change of APD_90_ following CCh application (atrial: *n* = 11/2, ventricular: *n* = 10/2). (*E*) Representative voltage-clamp recordings of inward-rectifier K^+^ current current in single iPSC-aCM (left) and iPSC-vCM (right) before (baseline, I_K1_) and after CCh application, revealing the acetylcholine-activated inward-rectifier K^+^ current (I_K,ACh_) in iPSC-aCM. (*F*), I_K1_ measured at −100 mV (atrial: *n* = 77/6, ventricular: *n* = 31/4). (*G*) Concentration-response curves for CCh-mediated activation of I_K,ACh_ defined as CCh-dependent increase of inward-rectifier-K^+^-current amplitude at −100 mV in iPSC-aCM (*n* = 7–77/6) and iPSC-vCM (*n* = 143/1). (*H*), Voltage-clamp protocol (0.5 Hz, top) and representative membrane current (I_M_) trace (bottom) of peak Na^+^ current (I_Na_) in iPSC-aCM (left) and iPSC-vCM (right). (*I*) Peak I_Na_ (atrial: *n* = 62/1, ventricular: *n* = 53/1). (*J*) Voltage-clamp protocol (0.5 Hz, top) and representative membrane current (I_M_) trace (bottom) of L-type Ca^2+^ current (I_Ca,L_) in iPSC-aCM (left) or iPSC-vCM (right). (*K*) Peak I_Ca,L_ (atrial: *n* = 66/2, ventricular: *n* = 176/2). (*L*) Representative flow cytometry analysis of iPSC-aCM staining for the atrial isoform (MLC2a, top) or the ventricular isoform (MLC2v bottom) of myosin light chain. Grey peaks represent the isotype control. Data are mean ± SEM. *P < 0.05, ***P < 0.001 vs. iPSC-aCM using unpaired Student’s *t*-test. n/*N* = number of iPSC-CM/differentiation.

iPSC-aCM and iPSC-vCM were generated by subtype-directed differentiation of iPSC from healthy donors as previously described (*Figure 1*).^27^ Temporal modulation of Wnt-signalling via small molecules (GiWi-protocol) stimulated cardiac differentiation. Atrial subtype specification was stimulated with 1 µmol/L retinoic acid between day 3 (d3) and day 6 of differentiation. CRISPR/Cas9 (clustered regularly interspaced short palindromic repeats/Cas9 nuclease)-mediated homologous recombination was used to insert the light-gated ion-channel f-Chrimson^26^ under the control of the CAG-promoter into the AAVS1 locus in a deeply characterized human iPSC line (TC-1133).^28,29^ This study was performed in line with the principles of the Declaration of Helsinki. All protocols were approved by the Ethics Committee of the University Medical Center Göttingen (No. 10/9/15 and 15/2/20). Informed consent was obtained from all participants and all research was performed in accordance with relevant guidelines and regulations.

### 2.2 Generation of engineered human myocardium (EHM)

**Figure 2:**
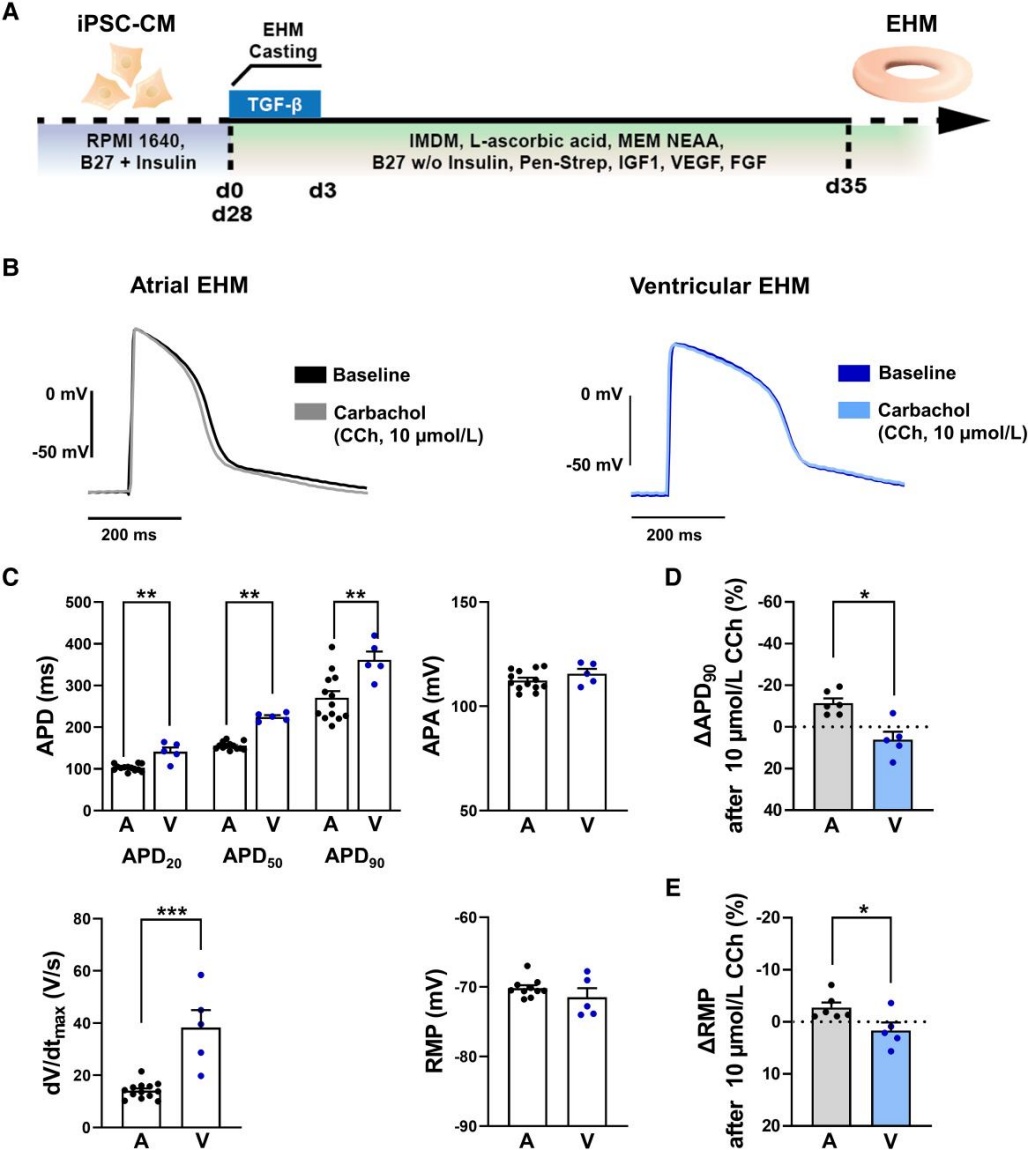
Electrophysiology of atrial (aEHM) and ventricular (vEHM) engineered human myocardium. (*A*) Schematic of the EHM culture procedure used in this study. (*B*) Representative action potentials (AP) elicited at 1 Hz in aEHM (*A*, left) and vEHM (V, right) before (baseline) and after application of the M-receptor agonist carbachol (CCh, 10 µmol/L). (*C*) AP duration at 20, 50, and 90% repolarization (APD_20_, APD_50_ and APD_90,_ top left), AP amplitude (APA, top right), upstroke velocity (dV/dt_max_, bottom left), and resting membrane potential (RMP, bottom right; atrial: *n* = 13/3, ventricular: *n* = 5/2). (*D*), Percentage change of APD_90_ following CCh application. (*E*), Percentage change of RMP following CCh application (atrial: *n* = 6/2, ventricular: *n* = 5/2). Data are mean ± SEM. *P < 0.05, **P < 0.01, ***P < 0.001 vs. aEHM using unpaired Student’s *t*-test or Welch’s *t*-test (*E*). n/*N* = number of recordings/EHM.

EHM was prepared as described previously (*Figure 2*).^23,30^ In brief: A mixture of iPSC-CM (70%), human fibroblasts (30%) and collagen was cast into a custom made 48-well plate (myrPlate, myriamed GmbH, Germany). EHM was constructed using iPSC-CM expressing f-Chrimson (see Supplementary material*online, Figure S1*) unless otherwise indicated. Spontaneous contractions were observed 3–5 days after the casting procedure. EHM was maintained in a serum-free medium (SFMM) containing IMDM with Glutamax, MEM Non-Essential Amino Acids Solution, 4% B27 without insulin (all Thermo Fisher Scientific), 200 µmol/L ascorbic acid 2-phosphate (Sigma-Aldrich), 100 ng/mL recombinant human IGF-1, 5 ng/mL recombinant human VEGF, 10 ng/mL animal-free recombinant human FGF-basic (all PeproTech). To allow for continuous real-time monitoring of force of contraction, EHM was prepared in an alternative engineering format embedded within custom-made culture vessels each directly coupled to an isometric force sensing device as previously described.^31^ All experiments were performed after a tissue culture period between 28 and 38 days.

### 2.3 Electrical and optical pacing of iPSC-CM and EHM

iPSC-aCM at d28 were plated onto Matrigel^®^-coated (1:120) 10 mm coverslips and maintained with a culture medium of RPMI 1640 with Glutamax, and 2% B27 (both Thermo Fisher Scientific) and maintained at 37°C, 5% CO_2_. Immediately before pacing, coverslips in a 6-well culture dish were submerged in a pacing medium of Medium 199 Glutamax (baseline Ca^2+^ concentration: 1.79 mmol/L) and 2% B27 (both Thermo Fisher Scientific). EHM mounted on stretchers were submerged in SFMM in a six-well culture dish. Electrical pacing was delivered to iPSC-aCM or aEHM at 1 or 3 Hz (always studied in parallel) for 24 h with biphasic 5 ms pulses via a C-pace EM culture stimulator (IonOptix, MA, USA).

Optical pacing was delivered via a custom-made optical pacing light emitting diode device placed above the transparent six-well culture plate lid. We quantified rheobase and chronaxie values (see Supplementary material*online, Figure S1*) to determine stimulation values for optical TP, which were set to 25% above contraction threshold (118 µW/mm² intensity, 5 ms).

Regular visual inspection at the start, after 8 h and at the end (24-h pacing) or every 24 h (7 day pacing) was undertaken to ensure cells and tissues were responsive to their supplied frequency at all investigated time-points. iPSC-aCM or aEHM were only included in this study if they passed this criterion. Seven-day contractile analysis during TP (see below) allowed for continuous real-time feedback of beating frequencies in response to pacing.

### 2.4 Optical AP recordings

Coverslips containing iPSC-CM were transferred to a 37 ± 0.5°C heated chamber with bath solution containing (in mmol/L): CaCl_2_ 2, Glucose 10, HEPES 10, KCl 4, MgCl_2_ 1, NaCl 140; pH = 7.35 adjusted with NaOH. Cells were loaded with 0.1 × FluoVolt (Thermo Scientific; 20 min loading). Intact cells were electrically field-stimulated at 1 Hz with 3–5 ms bipolar pulses at voltages ∼25% above the contraction threshold (normally between 10 and 30 V). AP were recorded optically at λ_ex_ = 470 nm and λ_em_ = 535 nm. Three AP from each iPSC-CM were ensemble averaged during offline analysis of AP parameters with Clampfit 10.7 (Molecular Devices, CA, USA).^32^

### 2.5 Patch-clamp measurements

Measurements of membrane currents were performed using conventional whole-cell ruptured-patch technique. Inward-rectifier K^+^ currents were recorded using the manual patch-clamp approach (Axopatch 200B, Molecular Devices) in iPSC-CM perfused with bath solution containing (in mmol/L): NaCl 120, KCl 20, MgCl_2_ 1, CaCl_2_ 2, glucose 10, HEPES 10; pH = 7.4 at 22–24°C. Borosilicate glass microelectrodes had tip resistances of 3–5 MΩ when filled with pipette solution (in mmol/L): K-aspartate 100, NaCl 10, KCl 40, Mg-ATP 5, EGTA 2, GTP-Tris 0.1, HEPES 10; pH = 7.4). Basal inward-rectifier K^+^ current was measured by applying a ramp pulse from −100 to +40 mV (0.5 Hz) and identified as Ba^2+^ (1 mmol/L)-sensitive current.^24,25,33,34^

I_Ca,L_ and I_Na_ recordings were performed using an automated patch-clamp system (SyncroPatch 384, Nanion Technologies GmbH, Germany) with thin borosilicate glass, single-aperture 384-well planar fixed-well chips.^13^ I_Ca,L_ was activated at 0.5 Hz using a voltage-step protocol with a holding potential of −80 mV and a 100 ms ramp pulse to −40 mV followed by a 100 ms test-pulse to +10 mV at 22–24°C. The internal solution contained (in mmol/L): EGTA 10, HEPES 10, CsCl 10, NaCl 10, CsF 110, pH 7.2 (with CsOH). The bath solution contained (in mmol/L): HEPES 10, NaCl 140, glucose 5, KCl 4, CaCl_2_ 2, MgCl_2_ 1; pH = 7.4 (with KOH) at 22–24°C. I_Na_ recordings were performed at 0.5 Hz using a voltage-step protocol with a holding potential of −100 and a 30 ms test pulse to −20 mV. Pipette solution contained (in mmol/L): EGTA 10, HEPES 10, KCl 10, NaCl 10, KF 110; pH = 7.2 (with KOH). Bath solution contained (in mmol/L HEPES 10, NaCl 140, glucose 5, KCl 4, CaCl_2_ 2, MgCl_2_ 1; pH = 7.4 (with KOH) at 22–24°C.

### 2.6 Sharp-electrode AP recordings

AP were recorded at 1 Hz in EHM using a Sec-05-X amplifier (npi Electronic GmbH, Germany) in voltage follower mode as previously described.^35^ Glass-microelectrodes filled with 3 mmol/L KCl had tip resistances of 30–40 MΩ.

### 2.7 Molecular analysis

The messenger RNA (mRNA) levels of key ion channel proteins were measured by real-time PCR using standard protocols.^23,36^ Flow cytometry analysis of iPSC-aCM was undertaken with antibodies against the atrial- (MLC2a) and the ventricular (MLC2v) isoform of myosin light chain using the BD Accuri™ C6 plus system flow cytometer (BD Biosciences, CA, USA).

### 2.8 Statistical analysis

Summarized data are reported as mean ± standard error of the mean (SEM) unless otherwise specified. Continuous data with a sample size *n* ≥ 20 were assumed to be normally distributed (central limit theorem). Data with the sample size between *n* = 10–20 were tested for normality using the Shapiro-Wilk test. Normally distributed data were compared using unpaired two-tailed Student’s *t*-test. Non-normally distributed data and all data sets with *n* < 10, were compared using the Mann–Whitney *U* test. Differences between data sets of unpaired data with unequal variances (F-test) were evaluated using Welch’s *t*-test.

## 3. Results

### 3.1 Cardiomyocytes and EHM with distinct atrial properties derived from human iPSC

Spontaneous beating frequency was higher in atrial compared to ventricular iPSC-CM (see Supplementary material*online, Figure S2*) as previously reported.^11^ In order to prevent frequency-dependent bias on AP parameters and ion channel properties, electrophysiological experiments were performed at defined stimulation frequencies throughout the study. We first assessed optical AP characteristics of intact, single atrial and ventricular iPSC-CM under 1 Hz field stimulation. As anticipated, APD at 50 and 90% repolarization (APD_50_, APD_90_) was shorter in iPSC-aCM compared to iPSC-vCM (*Figure 1B* and *C*). In native myocardium, vagal nerve stimulation shortens the atrial, but not ventricular AP.^37^ Accordingly, application of the muscarinic-receptor (M-receptor) agonist carbachol (CCh, 2 µmol/L) resulted in AP shortening in atrial, but not in ventricular iPSC-CM (*Figure 1B* and *D*).

AP shortening in response to M-receptor stimulation is mediated through activation of acetylcholine-activated inwardly rectifying K^+^ channels (I_K,ACh_).^24,25,38,39^ In agreement with this observation, mRNA levels of the corresponding channel subunit Kir3.1 (*KCNJ3*) were two-times higher in atrial vs. ventricular iPSC-CM (see Supplementary material*online, Figure S3*). In order to directly measure I_K,ACh_ in iPSC-CM, we employed a previously described patch-clamp depolarizing ramp protocol.^25,40^ Representative recordings in the absence of M-receptor agonists showed typical inward-rectifier properties in response to the depolarizing ramp-protocol with high conductance in the inward branch, which represents basal inward-rectifier K^+^ current I_K1_ (*Figure 1E*). As expected from reports on native human atrial and ventricular cardiomyocytes, I_K1_ was about 40% smaller in atrial vs. ventricular iPSC-CM (*Figure 1F*).^41,42^ However, only in iPSC-aCM did the application of CCh result in an increase in total current density (labelled I_K, ACh_) in a concentration-dependent manner (-lgEC_50_ = 6.24 ± 0.7 mol/L [571 nmol/L]; *Figure 1E* and *G*, Supplementary material*online, Figure S4*). In addition, despite the continuous presence of the M-receptor agonist, in iPSC-aCM I_K,ACh_ decreased from a ‘peak’-value to a ‘quasi-steady state’ (QSS) level within two minutes in a biphasic manner (see Supplementary material*online, Figure S4*). The QSS/Peak ratio (0.55 ± 0.03, *n* = 77) was similar to our earlier reports using native human atrial cardiomyocytes indicating comparable underlying mechanisms.^24,25^ Apart from canonical M-receptor mediated activation of I_K,ACh_, adenosine receptors can also activate cardiac I_K,ACh_ channels. Accordingly, adenosine activated K^+^ channels in iPSC-aCM in a concentration-dependent manner (-lgEC_50_ = 6.04 ± 0.4 mol/L [898 nmol/L]), whereas no effect was observed in iPSC-vCM (see Supplementary material*online, Figure S4*). Amplitudes of both I_Na_ (*Figure 1H* and *I*) and I_Ca,L_ (*Figure 1J* and *K*) were smaller in iPSC-aCM, with the latter potentially contributing to the shorter atrial APD (see Supplementary material*online, Figure S5*). Functional characterization of iPSC-aCM was supplemented with molecular analysis indicating minimal (2.6%) contamination of iPSC-vCM in the atrial cellular cohort. This was also confirmed by high throughput functional screening of I_K,ACh_ (see Supplementary material*online, Figure S6*).

In order to generate a multicellular, matured heart muscle environment, we next generated EHM from iPSC-aCM (aEHM) and iPSC-vCM (vEHM) (*Figure 2A*). Compared to vEHM, aEHM had a shorter APD (*Figure 2B* and *C*) and a smaller AP upstroke velocity. Application of 10 µmol/L CCh elicited significant shortening of APD_90_ and hyperpolarization of the RMP only in aEHM, not in vEHM. Taken together, this indicates the atrial phenotype is conserved in aEHM. Spontaneous beating has previously been shown to be faster in aEHM compared to vEHM,^11^ and is shown in Supplementary material*online, Video S1* and *Video S2*, respectively.

### 3.2 AP shortening and reduced I_Ca,L_ in iPSC-aCM and aEHM subjected to 24 h TP

**Figure 3:**
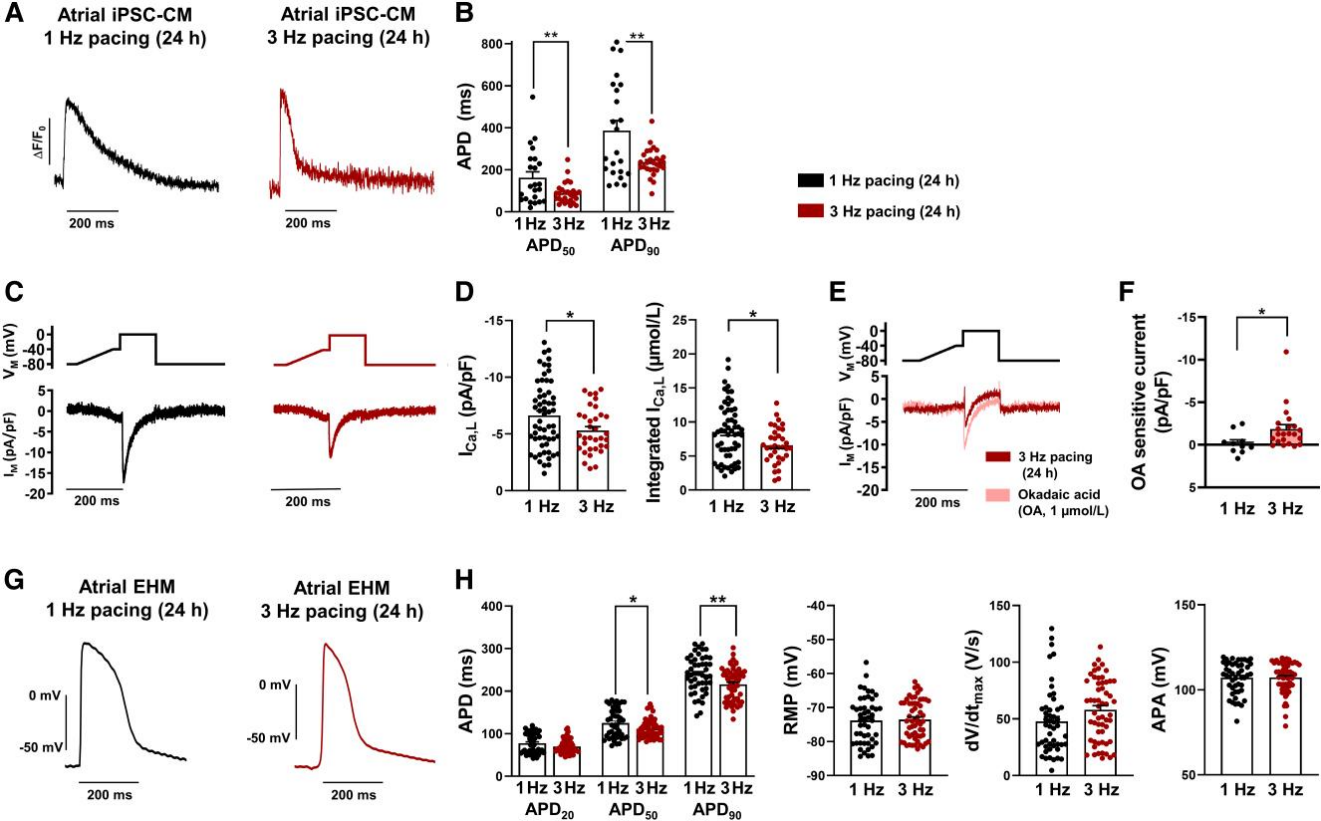
Twenty-four hour TP-induced remodelling of cellular electrophysiology in atrial induced pluripotent stem cell-derived cardiomyocytes (iPSC-aCM) and atrial engineered human myocardium (aEHM). (*A*) Representative optical action potentials (AP) elicited at 1 Hz in intact single iPSC-aCM after 24 h electrical pacing at 1 Hz (left) or 3 Hz (right). (*B*) AP duration at 50 and 90% repolarization (APD_50_, APD_90_, 1 Hz: *n* = 23/3, 3 Hz: *n* = 28/3) (*C*), Voltage-clamp protocol (0.5 Hz, top) and representative membrane current (I_M_) trace (bottom) of L-type Ca^2+^ current (I_Ca,L_) in iPSC-aCM after 24 h electrical pacing at 1 Hz (left) or 3 Hz (right). (*D*), Peak I_Ca,L_ (left), integrated I_Ca,L_ expressed as estimated cytosolic Ca^2+^ influx (right; 1 Hz: *n* = 23/4, 3 Hz: *n* = 28/4). (*E*), Voltage-clamp protocol (0.5 Hz, top) and representative membrane current (I_M_) trace (bottom) of I_Ca, L_ in 24 h 3 Hz paced iPSC-aCM before and after application of type 1 and type 2A phosphatase inhibitor okadaic acid (OA, 1 µmol/L). (*F*), Change in peak I_Ca,L_ following OA application (1 Hz: *n* = 11/1, 3 Hz: *n* = 22/1). (*G*) Representative AP were elicited at 1 Hz in aEHM after 24 h optical pacing at 1 Hz (left) or 3 Hz (right). (*H*) APD at 20, 50, and 90% repolarization (APD_20_, APD_50_ and APD_90,_ left), resting membrane potential (RMP,middle left), upstroke velocity (dV/dt_max_, middle right), AP amplitude (APA, right; 1 Hz: *n* = 49/14, 3 Hz: *n* = 61/15). Data are mean ± SEM. *P < 0.05, **P < 0.01 vs. 1 Hz using unpaired Student’s *t*-test or Welch’s *t*-test (*F*). n/*N* = number of iPSC-CM/differentiation or number of recordings/EHM.

In AF patients, sustained atrial tachycardia leads to electrical and structural remodelling that promotes chronification of AF.^1^ We therefore tested whether iPSC-aCM are also prone to develop electrical remodelling in response to *in vitro* TP. In agreement with our hypothesis, 24-h TP with 3 Hz to simulate atrial tachycardia^38^ resulted in shortening of the APD_50_ and APD_90_ in iPSC-aCM in comparison to controls subjected to 1 Hz pacing (*Figure 3A* and *B*). I_Ca, L_ amplitude was significantly reduced after 24-h TP (*Figure 3C* and *D*), suggesting that reduced depolarizing I_Ca,L_ could play a role in TP-dependent APD shortening (see Supplementary material*online, Figure S5*). TP-induced remodelling of I_Ca,L_ was independent of recording temperature (see Supplementary material*online, Figure S7*). Total Ca^2+^ influx was estimated by quantifying the area under the curve of the I_Ca,L_.^43^ Ca^2+^-influx was reduced by 23% in iPSC-aCM subjected to TP, which is considered a protective mechanism to limit detrimental Ca^2+^ overload during TP.^24,38^ We therefore hypothesized that TP-induced Ca^2+^ overload may be an important elicitor of electrical remodelling in iPSC-aCM. Accordingly, TP-induced AP shortening was absent following pacing in a low Ca^2+^ environment pointing to a Ca^2+^-mediated mechanism underlying TP-induced electrical remodelling in iPSC-aCM (see Supplementary material*online, Figure S8*). Hypophosphorylation of L-type Ca^2+^ channels has been consistently suggested as a potential contributor to I_Ca,L_ downregulation by various studies in samples from AF patients.^5^ In order to test whether reduced phosphorylation of I_Ca,L_ channels may also contribute to I_Ca,L_ downregulation in our model, we investigated the effects of type 1 and type 2A phosphatase inhibitor okadaic acid (OA, 1 µmol/L) on I_Ca,L_ in iPSC-aCM subjected to 24 h TP. Similar to observations in atrial cardiac myocytes from patients with AF, OA increased I_Ca,L_ amplitude only in iPSC-aCM paced at 3 Hz, not in the 1 Hz paced group pointing to an altered phosphorylation-dependent regulation of I_Ca,L_. (*Figure 3E* and *F*).

Following 24-h optical TP of aEHM, significant shortening of APD_50_ and APD_90_ was observed, but no TP-dependent effects were detected in RMP, AP upstroke velocity and AP amplitude (APA) (*Figure 3G* and *H*). This was also reflected in electrical pacing of aEHM (see Supplementary material*online, Figure S9*). The extent of electrical remodelling and AP shortening in aEHM was not altered by arrhythmogenic pacing, in which an irregular frequency is applied deviating with 50% variability from a 3 Hz mean (see Supplementary material*online, Figure S10*). TP-induced remodelling involving reduced I_Ca,L_ and a shorter AP was also observed in iPSC-vCM and vEHM, respectively, (see Supplementary material*online, Figure S11*) aligning with a previous study.^22^ This indicates that I_Ca,L_ downregulation is a common mechanism to prevent Ca^2+^ overload in response to tachycardia.

### 3.3 Response to vagal neurotransmitters is impaired in iPSC-aCM and aEHM subjected to 24 h TP

**Figure 4:**
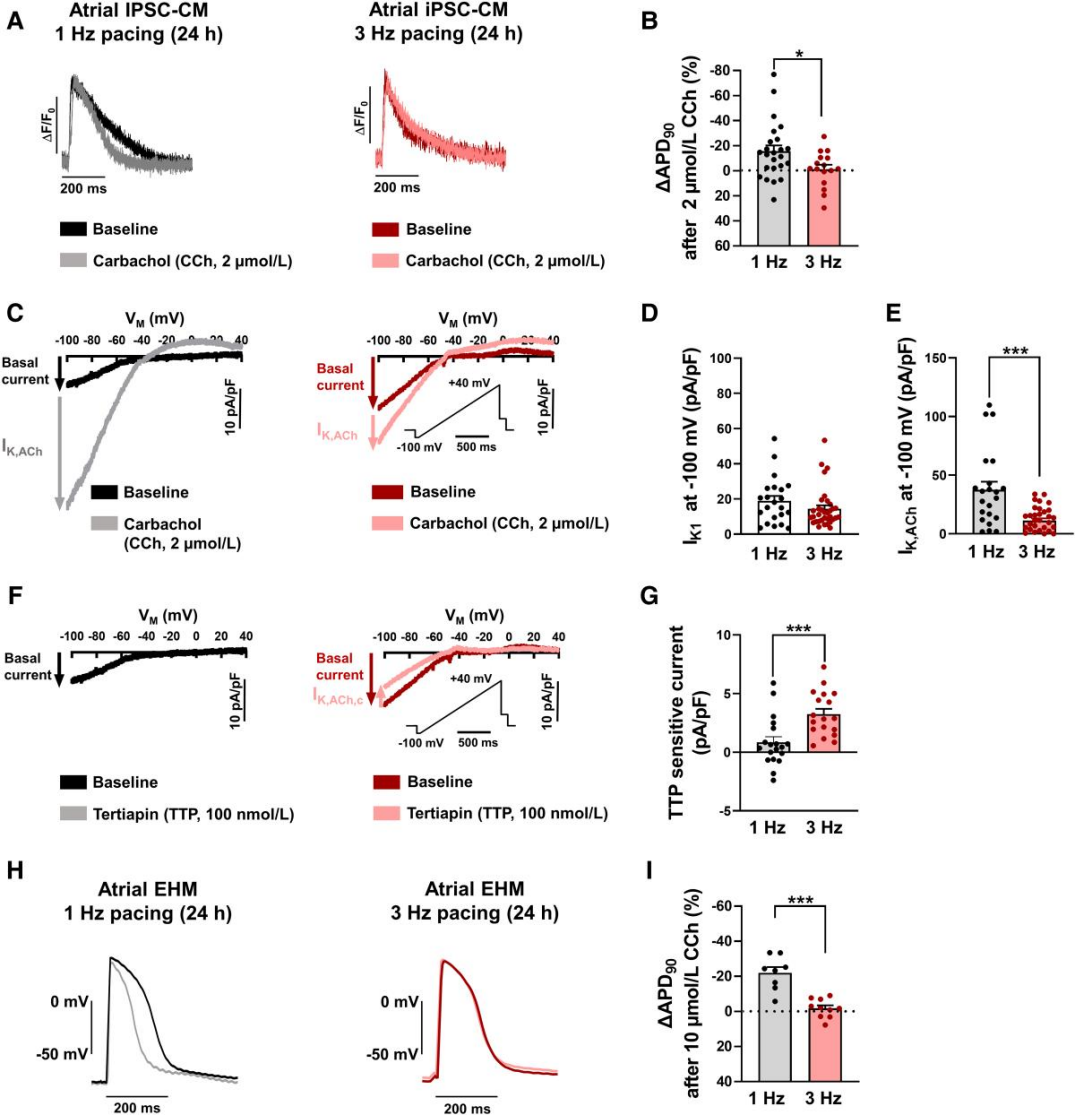
Twenty-four hour TP-induced remodelling of inward-rectifier K^+^ currents in atrial induced pluripotent stem cell-derived cardiomyocytes (iPSC-aCM) and atrial engineered human myocardium (aEHM). (*A*), Representative optical action potentials (AP) elicited at 1 Hz in intact single iPSC-aCM after 24 h electrical pacing at 1 Hz (left) or 3 Hz (right) before (baseline) and after application of the M-receptor agonist carbachol (CCh, 2 µmol/L). (*B*), Percentage change of AP duration at 90% repolarization (APD_90_) following CCh application (1 Hz: 26/4, 3 Hz: *n* = 16/3). (*C*), Representative voltage-clamp recordings of inward-rectifier K^+^ current in single iPSC-aCM after 24 h electrical pacing at 1 Hz (left) or 3 Hz (right) before (baseline, I_K1_) and after CCh application, revealing the acetylcholine-activated inward-rectifier K^+^ current (I_K,ACh_). (*D*), Peak I_K1_ measured at −100 mV. E, Peak I_K,ACh_ measured at −100 mV (*D*, *E*: 1 Hz: *n* = 22/2, 3 Hz: *n* = 34/2). (*F*), Representative voltage-clamp recordings of basal inward-rectifier K^+^ current in single iPSC-aCM after 24 h electrical pacing at 1 Hz (left) or 3 Hz (right) before (baseline) and after application of selective I_K,ACh_ blocker tertiapin (TTP, 100 nmol/L). (*G*), Change in basal current at −100 mV following TTP application, defined as agonist independent constitutive I_K,ACh_ (I_K,ACh,c_; 1 Hz: *n* = 19/2, 3 Hz *n* = 18/2). (*H*), Representative AP elicited at 1 Hz in aEHM after 24 h optical pacing at 1 Hz (left) or 3 Hz (right) before and after CCh application (10 µmol/L). (*I*), Percentage change of APD_90_ following CCh application (1 Hz: *n* = 8/5, 3 Hz: *n* = 10/5). Data are mean ± SEM. *P < 0.05, ***P < 0.001 vs. 1 Hz using paired Student’s *t*-test. n/*N* = number of iPSC-CM/differentiation or number of recordings/EHM.

APD shortening in response to application of M-receptor agonists such as CCh is a classical hallmark of atrial cardiomyocytes (*Figure 1B* and *D*). Furthermore, it is well known that this response is blunted in atrial cardiomyocytes from patients with AF.^24,25^ We, therefore, analysed AP response of iPSC-aCM subjected to 24-h TP to application of the M-receptor agonist CCh. Whereas CCh induced significant AP shortening in control iPSC-aCM paced at 1 Hz, this effect was abolished in the TP group (*Figure 4A* and *B*). To further unravel the underlying ion-channel remodelling, we quantified alterations of inward-rectifier K^+^ currents in response to 24-h TP. In the absence of CCh, inward-rectifier K^+^ current amplitude, which is mainly controlled by I_K1_ channels, was unaltered in response to 24-h TP (*Figure 4C* and *D*). In contrast, application of CCh resulted in a smaller current increase in the 24-h TP group pointing to reduced I_K,ACh_ current amplitude (*Figure 4C, E*), resembling AF-associated remodelling. Importantly, in iPSC-aCM subjected to TP, I_K,ACh_ recovered from TP-induced remodelling within additional 24-h normofrequent pacing at 1 Hz (see Supplementary material*online, Figure S12*). AP response to CCh was also restored in TP iPSC-aCM following an additional 24 h of 1 Hz pacing (see Supplementary material*online, Figure S12*).

It has been shown that atrial cardiomyocytes from patients with AF develop agonist-independent constitutive I_K,ACh_ (I_K,ACh,c_), which has been suggested to contribute to AP shortening in these patients.^25,38,39^ Since I_K,ACh_ channels are not expressed in ventricular myocytes, I_K,ACh,c_ represents a potential atrial- and pathology-specific drug target. To unmask I_K,ACh,c_ in iPSC-aCM subjected to TP, we applied the selective I_K,ACh_ blocker tertiapin (TTP) (100 nmol/L). In the absence of M-receptor agonists, TTP reduced the basal inward-rectifier K^+^ current in the TP group, but was without effect in control iPSC-aCM subjected to 24-h pacing at 1 Hz (*Figure 4F* and *G)*. Both the reduced agonist-dependent I_K,ACh_ and the development of agonist-independent constitutive I_K,ACh_ as observed in iPSC-aCM subjected to 24-h TP are classical hallmarks of AF-associated remodelling. These features were absent in iPSC-aCM subjected to TP in low Ca^2+^ (0.42 mmol/L) conditions, suggesting that TP-induced remodelling of I_K,ACh_ in iPSC-aCM is Ca^2+^ dependent (see Supplementary material*online, Figure S8*).

CCh (10 µmol/L) application to aEHM subjected to 24 h optical normofrequent pacing at 1 Hz shortened APD and hyperpolarized RMP (*Figure 4H* and *I*, Supplementary material*online, Figure S13*). These effects are thought to be mainly mediated by activation of I_K,ACh_ as observed in myocytes isolated from aEHM (see Supplementary material*online, Figure S4*). In contrast, the response to CCh was completely blunted after 24-hour TP of aEHM pointing to impaired I_K,ACh_ channel activity (*Figure 4E* and *F*).

### 3.4 Molecular features of aEHM after 24 h TP

**Figure 5:**
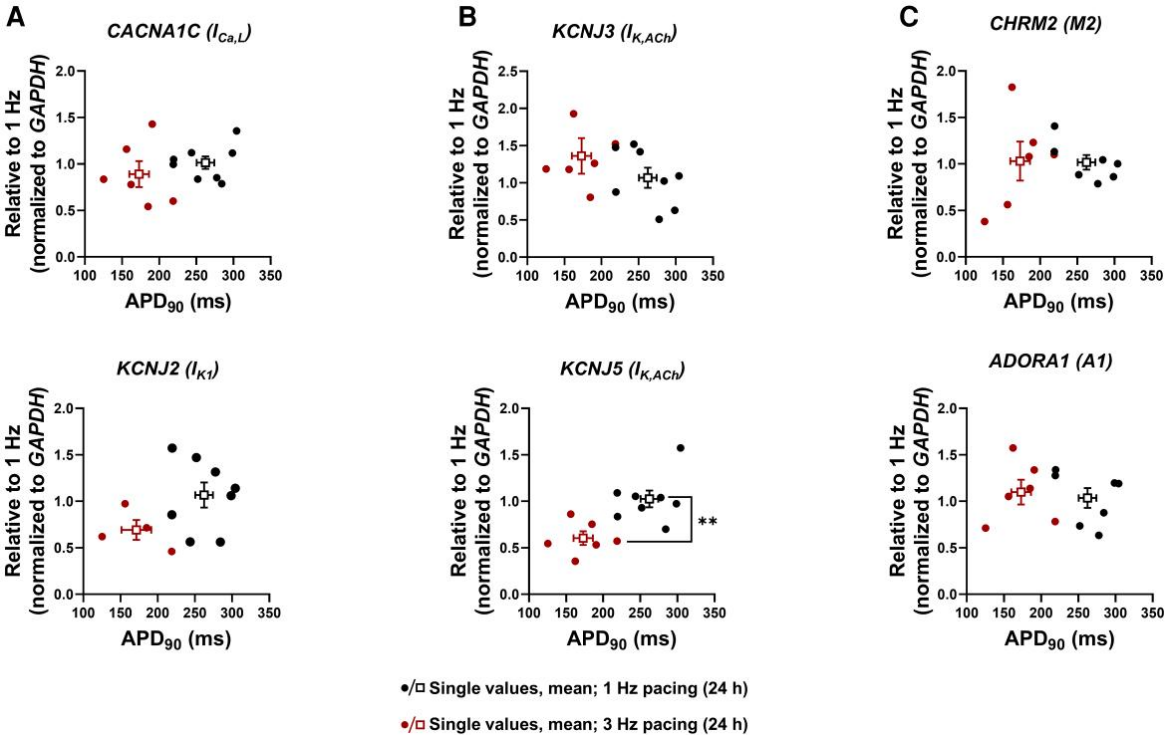
Gene expression of ionic channels and receptors in atrial engineered human myocardium (aEHM) subjected to 24-h optical TP. (*A*), mRNA levels of ion channels *CACNA1C* (I_Ca,L_, top) and *KCNJ2* (I_K1,_ bottom). (*B*), mRNA levels of I_K,ACh_ ion channel subunits *KCNJ3* (top) and *KCNJ5* (bottom). (*C*), mRNA levels of receptors *CHRM2* (M2 receptor, top) and *ADORA1* (A1 receptor, bottom). All values are plotted as single values against the corresponding action potential duration at 90% repolarization (APD_90_). Data are mean ± SEM. **P < 0.01 vs. 1 Hz using the Mann–Whitney *U* test.

In order to investigate the molecular basis of the observed electrophysiological phenotype and to determine whether alterations in ion-channel expression resemble those observed in patients with persistent AF, we quantified the expression of corresponding mRNA in aEHM subjected to optical TP (*Figure 5A*). Similar to persistent AF patients,^5^ expression of *CACNA1C* was unaltered suggesting that reduced I_Ca,L_ in aEHM subjected to TP is due to additional post-translational mechanisms such as hypophosphorylation (*Figure 3E* and *F*). In accordance with unaltered I_K1_ current in iPSC-aCM subjected to TP (*Figure 3C* and *D*), TP was without effect on the corresponding mRNA (*KCNJ2*).

In atrial myocytes, the I_K,ACh_ channel is a heterotetrameric complex composed of Kir3.1 and Kir3.4 subunits. TP-induced remodelling reduced the expression of *KCNJ5* mRNA encoding for Kir3.4 but had no effect on *KCNJ3* mRNA encoding for Kir3.1. Therefore, similar to observations in patients with persistent AF,^34^ impaired CCh response in aEHM subjected to TP appears to be mainly due to downregulation of the Kir3.4 channel subunit with less or absent reduction in Kir3.1 expression, respectively (*Figure 5B*). Expression of M-receptor subtype 2 (M2) and adenosine receptor (A1), which mediate activation of I_K,ACh_ in response to CCh and adenosine, respectively, was unaltered by TP (*Figure 5C*). This is in accordance with previous publications showing unaltered M2-receptor expression in patients with AF,^44^ suggesting that in both models reduced expression of I_K,ACh_ channels together with development of constitutive activity are the major contributors to reduced activation of I_K,ACh_ in response to CCh.

### 3.5 Seven-day Continuous TP of iPSC-aCM and aEHM reveals RMP hyperpolarization and I_K1_ upregulation

A major characteristic of AF is its chronically progressive nature. Whereas AF initially occurs as self-terminating episodes, i.e. paroxysmal AF, later stages of AF are characterized by the continuous persistence of the arrhythmia. Persistent AF is classified when episodes last longer than 7 days. The clinical progression of the arrhythmia is associated with progression of electrical remodelling.^2^

**Figure 6:**
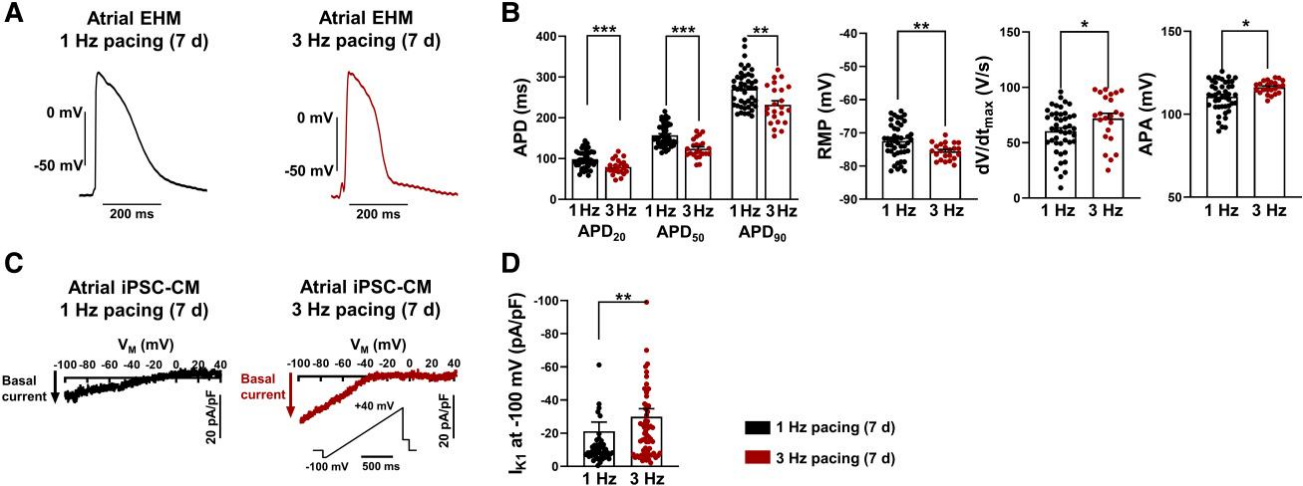
Prolonged (7-day) TP-induced remodelling of cellular electrophysiology in atrial engineered human myocardium (aEHM) and atrial induced pluripotent stem cell-derived cardiomyocytes (iPSC-aCM). (*A*), Representative action potentials (AP) elicited at 1 Hz in aEHM after 7 days of optical pacing at 1 Hz (left) or 3 Hz (right). (*B*), AP duration at 20, 50, and 90% repolarization (APD_20_, APD_50_ and APD_90,_ left), resting membrane potential (RMP, middle left), upstroke velocity (dV/dt_max_, middle right), AP amplitude (APA, right; 1 Hz: *n* = 45/8, 3 Hz: *n* = 23/9). (*C*) Representative voltage-clamp recordings of basal inward-rectifier K^+^ current (I_K1_) current in iPSC-aCM after 7 day of optical pacing at 1 Hz (left) or 3 Hz (right). (*D*) Peak I_K1_ measured at −100 mV (1 Hz: *n* = 49/1, 3 Hz: *n* = 64/1). Data are mean ± SEM. *P < 0.05, **P < 0.01, ***P < 0.001 vs. 1 Hz using paired Student’s *t*-test. n/*N* = number of iPSC-CM/differentiation or number of recordings/EHM.

Prolonged optical TP for 7 days resulted in a marked decrease of APD_50_ and APD_90_ in aEHM (*Figure 6A* and *B*). In addition, 7-day TP led to hyperpolarization of the RMP and an increase in APA. Both alterations were absent after short-term 24-h TP. Hyperpolarization of the RMP represents another hallmark of AF-associated remodelling which is likely due to I_K1_ upregulation.^24,35^ Indeed, basal inward-rectifier current was significantly higher in iPSC-aCM subjected to 3 Hz TP for 7 days compared to iPSC-aCM paced at 1 Hz for 7 days (*Figure *6*C* and *D*). The hyperpolarized RMP and increased I_K1_ observed after 7-day, but not after 24-h, TP suggest a different time-course in electrical remodelling in response to TP. Finally, AP response to CCh was blunted in aEHM after 7-day TP (see Supplementary material*online, Figure S14*).

### 3.6 Reduced contractility in aEHM after continuous 7-day TP

**Figure 7:**
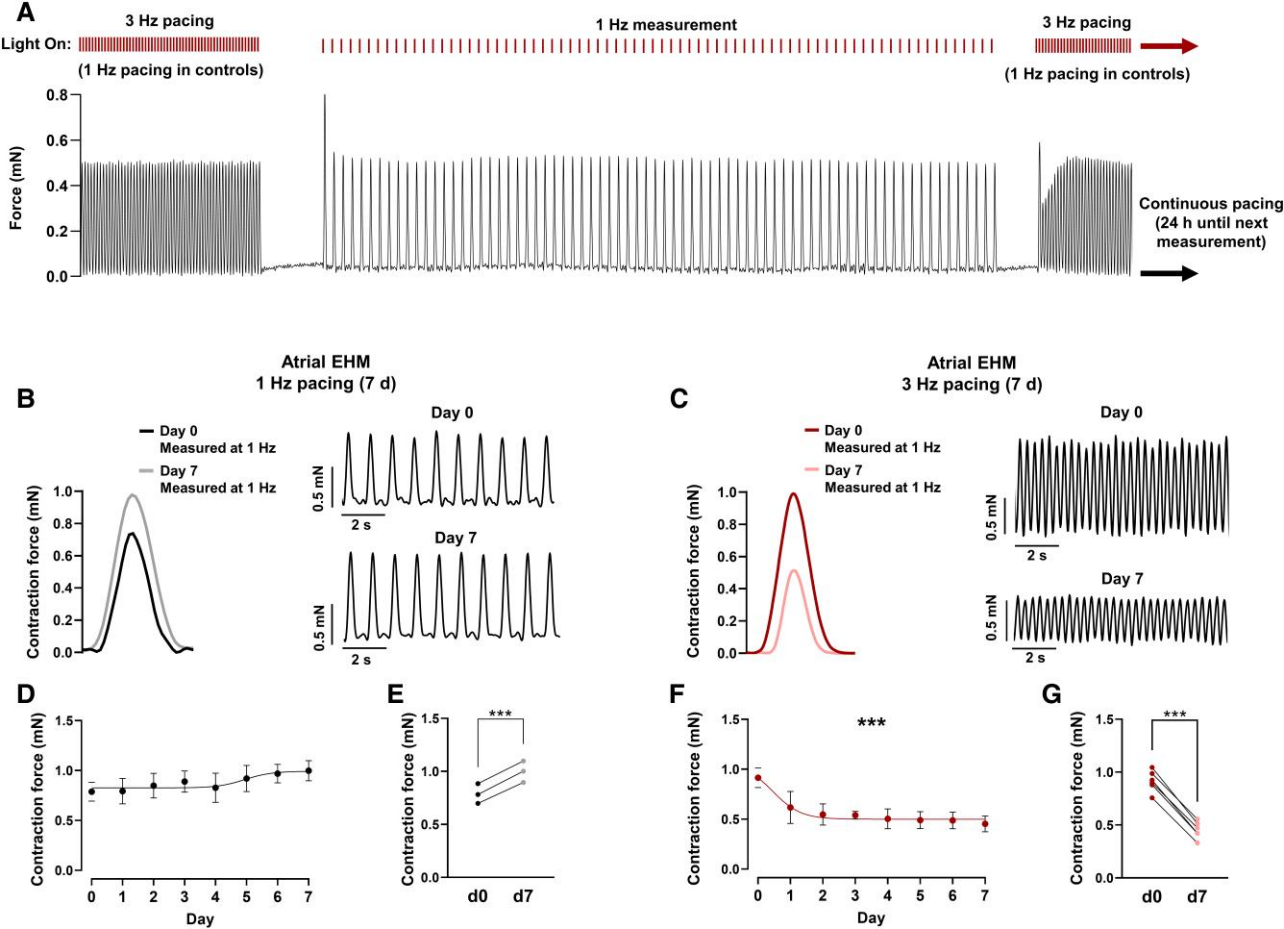
Prolonged (7-day) TP-induced contractile dysfunction in atrial human engineered myocardium (aEHM). (*A*) Representative partial trace of aEHM contraction as it is continuously optically stimulated at 3 Hz for 7 days. Contractile measurements were taken every 24 h at a window of 1 Hz pacing, of which a representative example is shown. (*B*) Representative average contractile signals from aEHM during 7 day 1 Hz optical pacing on day 0 and day 7 (left), representative partial traces of aEHM contraction on day 0 (top right) and day 7 (bottom right) of 1 Hz pacing. (*C*) Representative average contractile signals from aEHM tissue during 7 day 3 Hz optical pacing on day 0 and day 7 (left), representative partial traces of aEHM contraction on day 0 (top right) and day 7 (bottom right) of 3 Hz pacing. (*D*) Time course of contractile function of aEHM optically paced for 7 d at 1 Hz (*n* = 3). (*E*) Change in contractile force after 7 day of optical pacing at 1 Hz. Single point data extracted from (*D*). (*F*) Time course of contractile function of aEHM optically paced for 7 day at 3 Hz (*n* = 6). (*G*) Change in contractile force after 7 day of optical pacing at 3 Hz. Single point data extracted from (*F*). Data are mean ± SEM. ***P < 0.001 vs. 1 Hz using paired Student’s *t*-test (*E*, *G*) or an extra sum of squares F test to compare fitted sigmoidal curves (*D*, *F*). *n* = number of EHM.

aEHM were maintained inside force sensing culture vessels which were optically stimulated for 7 days at 1 Hz or 3 Hz. Force of contraction was measured at 1 Hz every 24 h (*Figure 7A*). Seven-day TP significantly reduced the force of contraction in comparison to control tissues stimulated at 1 Hz (*Figure 7B–G*). A clear reduction in force of contraction after only 1 day of TP is consistent with the decreased I_Ca,L_ observed after only 24-h TP in iPSC-aCM (*Figure 3C* and *D*) which could potentially influence the contractile properties of the cells. Furthermore, the reduction in force of contraction after 7 days of TP was reversible after a further 7 days of 1 Hz pacing (see Supplementary material*online, Figure S15*), suggesting that the initial contractility reduction during TP is unlikely to be caused by irreversible structural alterations such as increased apoptosis or fibrosis.

## 4. Discussion

In this study, we tested the hypothesis that iPSC-aCM and aEHM undergo electrical remodelling under TP, resembling phenotypic changes observed in patients with AF. We found that 24-h TP is sufficient to induce AP shortening in iPSC-aCM and aEHM, which is likely due to reduced I_Ca,L_ amplitude. In addition, 24-h TP blunted the response to CCh in both iPSC-aCM and aEHM, which is compatible with reduced agonist-dependent I_K,ACh_ activation and development of agonist-independent constitutive I_K,ACh_ activity (I_K,ACh,c_) in patients with AF. In contrast, 24-h TP was not sufficient to induce RMP hyperpolarization, which was observed after 7-day TP only, pointing to time-dependent development of specific hallmarks of electrical remodelling. Our data clearly demonstrate that several major hallmarks of AF-associated remodelling can be reproduced in iPSC-aCM and aEHM by electrical and optical TP.

### 4.1 AF-associated electrical remodelling

It is well known that AF is a progressive disease.^1,2^ Initially, AF often occurs as paroxysmal AF when episodes last less than 7 days and terminate spontaneously. However, with increasing duration of the rhythm disturbance, AF proceeds to become ‘persistent’ when conversion to sinus rhythm can only be achieved with pharmacological or electrical interventions, and ‘permanent’ when no further attempts to restore sinus rhythm are considered clinically useful.^45^ The progression of AF to more severe stages makes treatment of the arrhythmia in particular difficult. For many years there has been hope that unravelling the mechanisms underlying AF progression may lead to safer and more effective therapeutic rhythm control strategies.^1,46^

Wijffels *et al.* first demonstrated in a goat model that experimentally maintained AF alters atrial electrophysiology in a way that promotes persistence of the arrhythmia, thereby demonstrating that ‘AF begets AF’.^47^ In particular, the authors demonstrated that atrial TP led to shortening of the effective refractory period and coined the term ‘electrical remodelling’ to describe AF-promoting changes caused by AF itself.

In patients, decreased I_Ca,L_ and increased inward-rectifier K^+^ current I_K1_ are major hallmarks of electrical remodelling that lead to APD shortening and hyperpolarization of the RMP, respectively.^3–5,35,48,49^ In addition, reduced amplitude of the acetylcholine-activated inward-rectifier K^+^ current I_K,ACh_ and development of its agonist independent constitutive activity represent well-accepted components of electrical remodelling in patients with AF.^24,25,33,38^

It is believed that the very rapid atrial activity in AF represents a major driver for electrical remodelling.^21,24,38^ In fact, remodelling induced by AF is virtually indistinguishable from that produced by electrical tachycardia, and electrical TP of animal models has become one of the most accepted approaches to study mechanisms underlying AF-associated remodelling.^6,7^ TP-induced remodelling has since been demonstrated in dogs,^38,39,50^ sheep,^51,52^ rabbits,^53^ goats^47^ and pigs,^54^ where application of TP between 1 and 2 weeks leads to shortening of both effective refractory period and APD causing increased vulnerability to AF induction and increased arrhythmia persistence. Similar to AF patients, atrial myocytes isolated from those models show reduced I_Ca,L_, increased I_K1_ and reduced agonist-dependent I_K,ACh_ as classical hallmarks of electrical remodelling.

In addition to the reduced activation of I_K,ACh_ by M-receptor agonists, increased opening of agonist-independent constitutively active I_K,ACh_ channels is a major characteristic of electrical remodelling in AF patients and animal models of AF.^24,25,33,38,39^ I_K,ACh,c_ contributes to the basal inward-rectifier K^+^ current in AF and can be unmasked using the selective I_K,ACh_ inhibitor TTP as shown in atrial myocytes from AF patients and animal models as well as in the present study (*Figure 4*).^55^ TTP also terminates AF without affecting ventricular electrophysiology, indicating that I_K,ACh,c_ may be a potentially interesting anti-arrhythmic target.^56^ Consistent with previous data,^24,38^ our experiments suggest that development of I_K,ACh,c_ involves Ca^2+^-dependent mechanisms since I_K,ACh,c_ was absent in response to TP in a low Ca^2+^ pacing environment (see Supplementary material*online, Figure S8*). As shown in dog atrial myocytes and patients with AF, the rate-induced Ca^2+^ overload is thought to activate calpain that cleaves cell proteins and leads to the breakdown of the I_K,ACh_ inhibitory protein kinase C alpha.^24,38^ Together with increased membrane translocation of the stimulatory protein kinase C epsilon, these mechanisms are likely to contribute to I_K,ACh_ remodelling in AF and TP models.^38,57^

### 4.2 iPSC-aCM and aEHM as AF models

Over the past few years, we have learnt a great deal about molecular mechanisms underlying the initiation and maintenance of AF, by studying right atrial biopsies from patients undergoing open heart surgery.^1,2,58^ Studying human atrial samples mainly allows for the bare description of the arrhythmogenic substrate, whereas interventions that modify development of an arrhythmogenic substrate can only be investigated to a very limited extent. Animal models allow for careful control of potential disease-modifying factors and are therefore useful tools for the exploration of mechanistic hypotheses.^6,7^ However, especially with respect to cellular electrophysiology and Ca^2+^ handling, animal models show substantial differences from the human atrium, which may explain why findings obtained in myocytes from animal models often cannot be reproduced in human atrial myocytes.^8^ There is hope that the development of human atrial tissue models based on iPSC-aCM represents a valuable tool to bridge the translational gap between basic arrhythmia research and clinical applications. In fact, a recently issued statement by the US Food and Drug Administration (FDA) emphasized that data from *in vitro* models may be increasingly accepted as pivotal evidence to support clinical trial applications.

In an attempt to promote subtype-directed differentiation, retinoic acid has been shown to regulate the fate specification of atrial vs. ventricular myocytes during cardiac differentiation of human pluripotent stem cells.^11,59,60^ Shorter APD is a consistent observation in atrial vs. ventricular iPSC-CM, which is mirrored by our findings.^11,12,60–62^ APD_50_ of iPSC-aCM are comparable to values of APD_50_ obtained in freshly isolated native human atrial myocytes from patients undergoing open heart surgery.^2,3^ Furthermore, aEHM generated from iPSC-aCM are enriched in atrial-specific markers [*NPPA*, *KCNJ3* and *GJA5*, myosin light chain 2A (*MYL7*)] whereas ventricular markers [myosin light chain 2 V (*MYL2*) and *IRX4*) are almost absent (*Figure 1*).^11^ mRNA of atrial-specific ion channels Kv1.5 (*KCNA5*) and Kir3.1 (*KCNJ3*), which meditate the ultra-rapid delayed rectifier K^+^ current I_Kur_ and the acetylcholine-activated inward-rectifier K^+^ current I_K,ACh_, respectively, show higher expression in iPSC-aCM than in iPSC-vCM (see Supplementary material*online, Figure S3*).^11^ In accordance, agonist-dependent I_K,ACh_ currents can be activated in atrial but not in ventricular iPSC-CM (*Figures 1* and *2*, Supplementary material*online, Figure S6*).^12,14,63^ Here we demonstrate that I_K,ACh_ channel properties were comparable to our previously reported I_K,ACh_ properties in native human atrial myocytes with respect to agonist-dependent activation and desensitization (*Figure 1*, Supplementary material*online, Figure S4*). In agreement with previous data reporting a reduced expression of Kir2.1 (*KCNJ2*) and Cav1.2 (*CACNA1C*) in atrial samples,^13,64^ we found smaller I_K1_ and I_Ca,L_ densities in iPSC-aCM (*Figure 1*). This was not fully recapitulated on an mRNA expression level with lower *KCNJ2* (*P* < 0.05), but higher *CACNA1C* (not significant; Supplementary material*online, Figure S3*).

In order to simulate atrial arrhythmias, multicellular preparations of iPSC-aCM have been investigated in 2D culture dishes^60^ and 3D engineered heart tissue constructs.^65^ Both models have been shown to be susceptible to various forms of re-entry, which could be terminated by application of Na ^+^ -channel blockers flecainide and vernakalant or electrical stimulation. Whereas these investigations represent an important proof-of-concept, they do not take AF-associated remodelling into account. In particular, specific atrial targets such as I_Kur_ are downregulated in AF^35^ whereas others develop in a disease specific manner such as I_K,ACh,c_, which is absent in healthy atria.^24,25,38^ Given that changes in ion channel function caused by electrical remodelling and AF persistence represent a major challenge in AF therapy development,^18,19^ electrical remodelling needs to be considered when implementing iPSC-aCM-based models of AF.

Electrical *in vivo* TP in animal models of AF can be applied for more than 4 weeks. However, electrical *in vitro* TP of atrial myocytes is only feasible for about 24 h. This limitation results from adverse Faradic reactions including oxidation of electrodes, generation of chlorine and hydroxyl radicals, and formation of hypochlorous acid and chlorate.^22,66^ To overcome this caveat, expression of Channelrhodopsin-2, a light-gated ion channel, using lentiviral-mediated transduction has been employed previously to allow long-term stimulation of EHM.^22,62^ However, due to strong desensitization properties of Channelrhodopsin-2, this approach does not allow continuous high-frequency stimulation since a recovery period of 15 s is required after 15 s of 3 Hz burst stimulation to allow channel recovery.^22^ This intermittent pacing may explain why, in a recent study using this approach, only limited remodelling was achieved in atrial EHM.^62^ To circumvent these difficulties and allow for long-term high-frequency pacing we used a novel Channelrhodopsin variant f-Chrimson with faster on/off kinetics and reduced desensitization properties.^26^ We generated an iPSC line expressing f-Chrimson using CRISPR/Cas9 genome editing technology to ensure that all atrial cells derived from this line express f-Chrimson. In contrast to lentiviral transduction methods, which have an efficacy of ∼25% according to previous publications,^22,62^ this approach further reduces the required light intensity to avoid desensitization during high pacing frequencies. Using this approach we were able to continuously apply 3 Hz pacing for 7 days and successfully induce electrical remodelling in aEHM.

Similar to consistent findings in patients with AF and animal models subjected to TP, here we show for the first time that 24-h TP is sufficient to induce electrical remodelling in iPSC-aCM characterized by APD shortening, downregulation of I_Ca,L_ and impaired activity of I_K,ACh_. Interestingly, 24-h TP of iPSC-vCM and vEHM also induced I_Ca,L_ downregulation and AP shortening, which is consistent with previous data^22^ and points to a ubiquitous mechanism to avoid cellular Ca^2+^ overload by limiting Ca^2+^ influx. Nevertheless, 24-h TP was not sufficient to induce upregulation of basal inward-rectifier K^+^ current I_K1_ and hyperpolarization of the RMP. Similarly, previous findings indicated that 24-h TP of isolated dog atrial myocytes led to AP shortening, reduced I_Ca,L_ amplitude and remodelling of I_K,ACh_ but without any effect on RMP, indicating unaltered I_K1_.^21,38,39^ In fact, we observed RMP hyperpolarization in aEHM after 1 week TP, which is likely to be caused by increased I_K1_, suggesting that longer periods of tachycardia are necessary for RMP hyperpolarization. This is consistent with our previous findings in atrial myocytes from patients with paroxysmal AF i.e. AF episodes lasting less than 1 week, showing unaltered amplitudes of basal inward-rectifier K^+^ currents^24,33^ whereas agonist-dependent I_K,ACh_ was already reduced.

We propose that the possibility to directly study electrical remodelling in human atrial myocytes will enable more rapid and accurate identification of the underlying mechanisms in a human-specific context. In addition, molecular mechanisms of agonist-independent I_K,ACh_ have been investigated in great detail,^24,34,38^ but translation into clinically available treatments have so far failed because currently available drug screening assays are based on non-cardiac cell lines or animal models.^24,25,38^ None of them completely represents the nature of constitutively active I_K,ACh_. Furthermore, although reduced I_K,ACh_ current has been detected in animal models of AF,^38,67^ downregulation of underlying Kir3.1 and Kir3.4 channel subunits was absent in animal models.^39^ In contrast, expression of Kir3.4 (*KCNJ5*), but not Kir3.1 (*KCNJ3*) mRNA was reduced in aEHM subjected to TP, which is in accordance with findings in AF patients, where protein expression of Kir3.4 is more strongly reduced compared to Kir3.1.^34^ These data indicate that reduced expression of I_K,ACh_ channels represents a human-specific mechanism in I_K,ACh_ remodelling. Furthermore, we describe a phosphorylation-dependent downregulation of I_Ca,L_ (*Figure 3*) in the absence of decreased Ca_V_1.2 (*CACNA1C*) expression (*Figure 5*) which has also been shown in patients with persistent AF.^5,68^ In contrast, animal models of AF-associated remodelling predominantly show a transcriptionally mediated reduction of Ca_V_1.2.^69–71^ This indicates another potential human-specific mechanism in response to atrial TP.

Similar to other common cardiovascular diseases, AF is a multifactorial disease with both environmental and genetic factors contributing to pathogenesis. In addition to familial AF, with early onset and clear hereditary patterns,^72^ genome-wide association studies (GWAS) have identified a plethora of common variants associated with AF.^73^ Most of those variants are located in non-coding regions of the genome with no clear path from gene to disease mechanism. Unravelling the underlying mechanisms will likely require large-scale functional screening assays that are able to reproduce major aspects of atrial electrophysiology and are susceptible to AF-associated electrical remodelling. In addition, it is assumed that genomic variability underlying AF also contributes to the variable responses of patients to antiarrhythmic therapy.^74^ Therefore, personalized atrial models based on iPSC-aCM may be useful for predicting patients’ individual drug response. Finally, it is presumable that gene variants associated with AF not only increase the susceptibility to AF induction but also facilitate the development of electrical remodelling thereby promoting AF maintenance in certain patients. Our present findings suggest that iPSC-aCM not only represents a valuable tool to study the impact of genetic variants on atrial electrophysiology and drug response, but they can also be employed to study whether certain variants promote or protect from electrical remodelling. Therefore, these models represent an important step towards personalized treatment of AF.

### 4.3 Potential limitations

It is important to be aware that there is no ‘perfect’ model of AF.^7^ The pathophysiology of AF in the individual patient is a complex function of underlying diseases, genetic predisposition and environmental factors. In fact, electrophysiological phenotypes differ from patients with long-term persistent AF,^3,24,25^ paroxysmal AF,^2,33,75^ postoperative AF^76^ or AF in patients with heart failure.^77^ Therefore, any model of AF only reproduces certain aspects of this complex phenotype and models need to be chosen based on the research question being asked. Accordingly, it is well known that iPSC-aCM represent a rather immature developmental stage. Immature subcellular structure, Ca^2+^-handling properties and the presence of depolarizing ion currents resulting in spontaneous activity need to be considered when using iPSC-based models.^78–80^ In addition, although expression of Kv1.5 channels is significantly higher in iPSC-aCM compared to iPSC-vCM,^11^ corresponding I_Kur_ currents^81^ are still small compared to native human atrial CM.^82,83^ This may explain why we were not able to detect a prolongation of APD_20_ in response to 3-Hz TP, which is supposed to represent an accepted hallmark of AF-associated remodelling caused by I_Kur_ downregulation.^1,2^ Nevertheless, human iPSC-CM present a readily available human model of cardiomyocytes which can be generated on demand in large quantities, making them a promising model to investigate electrophysiological abnormalities in patients.^11^

For technical reasons, high-throughput electrophysiological recordings of I_Ca,L_ (*Figures 1 and 3*) and inward-rectifier potassium currents (*Figure 6*) were performed at room temperature only.^13^ Whereas previously published data render temperature effects on I_K1_ and I_K,ACh_ unlikely,^33^ I_Ca,L_ amplitudes are clearly temperature dependent. However, additional experiments performed at 37°C using conventional patch-clamp confirmed our findings of clearly smaller I_Ca,L_ amplitude after 24 h electrical TP at 3 Hz compared to pacing at 1 Hz (see Supplementary material*online, Figure S7*).

## 5. Conclusions

In this study, we developed a new AF model based on iPSC-aCM and aEHM. We clearly demonstrate that electrical TP of iPSC-aCM and aEHM induces electrophysiological remodelling resembling the situation in patients with persistent AF. In particular, we observed shorter APD, reduced I_Ca,L_ and I_K,ACh_ and development of agonist independent constitutively active I_K,ACh_.

We furthermore introduced a new iPSC-line expressing fast Channelrhodopsin f-Chrimson. This allowed for novel continuous long-term *in vitro* TP of cardiac tissue in general and of aEHM in particular. Thereby we could demonstrate that various hallmarks of electrical remodelling develop at different time points with APD shortening, abnormal I_Ca,L_ and I_K,ACh_ activity being early events whereas RMP hyperpolarization could only be detected after 1 week.

Taken together, we believe that these models represent an important new tool for studying mechanisms underlying AF-associated electrical remodelling in humans and for the development of new patient-tailored antiarrhythmic therapy.

## Supplementary material

Supplementary material is available at *Cardiovascular Research* online.

## Supplementary Material

cvad143_Supplementary_Data

## Data Availability

All available data are incorporated into this article and its online supplementary material.

## Notes

Translational perspective Atrial fibrillation (AF) begets AF through electrical remodelling. Robust human-centric atrial models of AF-associated remodelling are limited. Through tachypacing of atrial subtype-specific induced pluripotent stem cell-derived cardiomyocytes and engineered human myocardium, we provide novel 2D and 3D *in vitro* models that replicate the hallmarks of classical AF-associated electrical remodelling. Optogenetic pacing uniquely enables continuous long-term tachypacing to investigate the time dependence of AF evolution, analogous to the clinical progression of paroxysmal to permanent AF. These models can be implemented in high-throughput screening platforms to facilitate humancentric, atrial-specific development of antiarrhythmic therapies.
