# Supplementary material for: Atrial fibrillation-associated electrical remodelling in human induced pluripotent stem cell-derived atrial cardiomyocytes: a novel pathway for antiarrhythmic therapy development
Source: Cardiovasc Res. 2023 Sep 7;119(16):2623–37. doi: 10.1093/cvr/cvad143 (PMC10730244; doi:10.1093/cvr/cvad143)
Supplement: cvad143_Supplementary_Data [file cvad143_supplementary_data.zip › 3Supplemental material_Seibertz_CVR_2023_FINAL.pdf]

**SUPPLEMENTAL MATERIAL**

# Atrial Fibrillation-Associated Electrical Remodelling in Human Induced Pluripotent Stem Cell-Derived Atrial Cardiomyocytes: A Novel Pathway for Antiarrhythmic Therapy Development

Fitzwilliam Seibertz[1,2,3,*], Tony Rubio[1,2,*], Robin Springer[1,2,*], Fiona Popp[1,2], Melanie Ritter[1,2], Aiste Liutkute,[1,2] Lena Bartelt,[1,2] Lea Stelzer[1,2], Fereshteh Haghighi[2,4], Jan Pietras[2,4], Hendrik Windel[2,4], Núria Díaz i Pedrosa[1,2,§], Markus Rapedius[5], Yannic Döring,[1,2] Richard Solano1[,2,4], Robin Hindmarsh[2,6], Runzhu Shi[7,2], Malte Tiburcy[1,2], Tobias Brügmann[7,2,3], Ingo Kutschka[2,4], Katrin Streckfuss-Bömeke[2,6,8], George Kensah[2,4], Lukas Cyganek[2,3,6], Wolfram H. Zimmermann[1,2,3,9,10,11], Niels Voigt[1,2,3]

[1]Institute of Pharmacology and Toxicology, University Medical Center Göttingen, Georg-August University Göttingen, Germany

[2]DZHK (German Center for Cardiovascular Research), partner site Göttingen, Germany

[3]Cluster of Excellence "Multiscale Bioimaging: from Molecular Machines to Networks of Excitable Cells" (MBExC), University of Göttingen, Göttingen, Germany

[4]Department of Cardiothoracic and Vascular Surgery, Georg-August-University Göttingen, Göttingen, Germany

[5]Nanion Technologies GmbH, Munich, Germany

[6]Clinic for Cardiology and Pneumology, University Medical Center Göttingen, Georg-August University Göttingen, Germany

[7]Institute for Cardiovascular Physiology, University Medical Center Göttingen, Göttingen, Germany

[8]Institute of Pharmacology and Toxicology, University of Würzburg, Würzburg, Germany

[9]German Center for Neurodegenerative Diseases (DZNE), Göttingen, Germany

[10]Fraunhofer Institute for Translational Medicine and Pharmacology (ITMP), Göttingen, Germany

[11]Campus-Institute Data Science (CIDAS), University of Göttingen, Germany

[§]current affiliation: Institute of Experimental Cardiology, Internal Medicine VIII, Heidelberg University, Heidelberg, Germany

**Running title:** AF-associated remodelling in atrial iPSC-CM

*The first three authors contributed equally to this study.

**Corresponding Author:**
Niels Voigt, Institute of Pharmacology and Toxicology, Robert-Koch-Straße 40, 37075 Göttingen, Germany, Tel.: 00495513965174, Fax: 00495513965169, E-mail: niels.voigt@med.uni-goettingen.de
ORCID ID: 0000-0001-8230-2341

# Table of Contents

A human model of atrial fibrillation featuring atrial iPSC derived cardiomyocytes .......... **Error! Bookmark not defined.**

Supplemental Methods ........ 3

Cardiac differentiation of human iPSC-CM ........ 3

Native human cardiac tissue samples ........ 4

Coverslip preparation ........ 4

Preparation of engineered human myocardium (EHM) ........ 4

Electrical pacing of atrial iPSC-CM and atrial EHM ........ 5

Optogenetic stimulation of TC1133_CAG-Chrimson-YFP iPSC derived cardiomyocytes ........ 5

Optical action potential recordings in iPSC-CM ........ 5

Voltage-clamp measurements of inward-rectifier $K^+$ currents ........ 6

Automated voltage-clamp measurements of $I_{Ca,L}$ and $I_{Na}$ ........ 6

Sharp microelectrode measurements ........ 8

Contractile force analysis ........ 8

FACs analysis ........ 9

Gene-expression analysis ........ 10

Supplemental Figures ........ 12

Supplemental Videos ........ 29

Supplemental References ........ 30

## Supplemental Methods

### *Cardiac differentiation of human iPSC-CM*

All protocols were approved by the Ethics Committee of the University Medical Center Göttingen (No. 10/9/15 and 15/2/20). Informed consent was obtained from all participants and all research was performed in accordance with relevant guidelines and regulations.

All iPSC lines in this study are registered in the human pluripotent stem cell registry (https://hpscreg.eu/). iPSC lines UMGi014-C clone 14 (isWT1.14) and RUCDRi002-A-46 clone 1 (TC-1133-CAG-Chrimson-YFP.1TC1133_CAG-Chrimson-YFP)[1] were differentiated into ventricular and atrial cardiomyocytes and cultured according to the classical GiWi protocol.[2,3,4] iPSC were cultured under feeder-free conditions with StemMACS™ iPS-Brew XF (Miltenyi Biotec) until confluency was reached. Mesoderm induction was initiated through 48-hour incubation with 4-5 µmol/L CHIR99021 in a 'Differentiation Medium' containing: RPMI 1640 with Glutamax (Thermo Fisher Scientific), 0.5 mg/ml human recombinant albumin, and 0.2 mg/ml L-ascorbic acid 2-phosphate (all Sigma-Aldrich). The day of CHIR99021 addition is referred to as day 0. After 2 days, cardiac differentiation was started by addition of 5 µmol/L IWP2 (Wnt Antagonist II, Merck) to the differentiation medium for 48 h.[3,4] For atrial subtype specification, 1 µmol/L retinoic acid (Sigma Aldrich) was added between day 3 and day 6.[5] Cardiac contraction is usually observed on day 7. From day 8 onwards, cells are cultured in a 'culture medium' containing RPMI 1640 with Glutamax, and 2% B27 (both Thermo Fisher Scientific) with medium changes every 2-3 days. On day 15, metabolic selection[6] was applied for 7 days to increase cardiomyocyte purity with a selection medium of RPMI 1640 without glucose (Thermo Fisher Scientific) supplemented with 0.5 mg/ml human recombinant albumin, 0.2 mg/ml L-ascorbic acid 2-phosphate, and 4 mmol/L lactate (all Sigma-Aldrich). After 7 days, medium was switched back to culture medium. On day 28, the iPSC-CM were plated on glass coverslips for isolated cell experiments or used for EHM preparation.

***Native human cardiac tissue samples***

Right atrial appendages were obtained from 5 patients in normal sinus rhythm undergoing cardiac surgery. Left ventricle tissue was obtained from 4 patients with severe aortic valve stenosis undergoing cardiac surgery for aortic valve replacement (Morrow resection). Excised tissue samples were snap-frozen in liquid nitrogen for biochemical studies. Experimental protocols were approved by the ethics committee of the University Medical Center Göttingen (No. 4/11/18). Each patient gave written informed consent.

***Coverslip preparation***

Wells containing iPSC-CM were washed twice with DPBS Ca/Mg$^{-/-}$ (Gibco/Thermo Fisher Scientific), and then incubated with Accutase® (Merck) for 10-15 min, until the cells were detached and dissociated. Digestion was stopped with 'recovery medium' containing culture medium supplemented with 1:2000 ROCK inhibitor Y27632 (REPROCELL Stemgent). Following centrifugation at 4°C at 100 g for 10 minutes, cells were resuspended in recovery medium, counted using a counting chamber (Neubauer improved, Paul Marienfeld GmbH & Co.KG) and plated on glass coverslips (10 mm diameter Menzel coverslip, ThermoFischer) pre-coated with 1:120 Matrigel® (Corning®) at a density of 1x10$^{3}$ cells/coverslip. iPSC-CM were incubated with recovery medium for 2 days and then switched to culture medium and maintained every 2-3 days. All iPSC-CM measurements were conducted between d35 and d43.

***Preparation of engineered human myocardium (EHM)***

Engineered human myocardium (EHM) was prepared according to published protocols.[7,8] A mixture of iPSC-CM (d28) and human foreskin fibroblasts (HFF-1) (70:30; 0.5x10$^{6}$ cells/EHM) was suspended in a collagen type I hydrogel (0.3 mg/EHM; Cat. #FS22024; Collagen Solution) supplemented with 2X RPMI (Thermo Fisher Scientific) in a serum-free maturation medium (SFMM) containing IMDM with Glutamax, MEM Non-Essential Amino Acids Solution, 4% B27 without insulin (all Thermo Fisher Scientific), 200 µmol/L Ascorbic acid 2-phosphate, 100 U/mL penicillin and 100 µg/mL streptomycin (all Sigma-Aldrich) 100 ng/mL Recombinant Human IGF-1, 5 ng/mL Recombinant Human VEGF, 10 ng/mL Animal-Free Recombinant Human FGF-basic (all PeproTech) and finally pH-neutralized by drop wise addition of 0.1 mol/L NaOH. The mixture was poured into individual wells (180 µL/EHM) of a 48-well myrPlate (uniform-TM5,

myriamed GmbH) and incubated at 37 °C, 5% $CO_2$ for 1 h to facilitate uniform EHM consolidation. Subsequently, a SFMM with 0.5 ng/mL TGF-β1 (PeproTech) was added to each well. This medium was changed daily for 3 days. Thereafter, SFMM without TGF-β1 was used and exchanged every second day until EHM measurement. All culture was conducted at 37 °C, 5% $CO_2$. EHM contractions are typically observed 3-5 days after casting. All experiments were performed 28 to 38 days after casting.

***Electrical pacing of atrial iPSC-CM and atrial EHM***

For electrical tachypacing, glass coverslips containing iPSC-CM were gently transferred to a 6-well plate filled with 6 mL cardiomyocyte pacing medium (Medium 199 [$Ca^{2+}$ concentration: 1.79 mmol/L] with Glutamax, and 2% B27 supplement [both Thermo Fisher Scientific]). After 28 to 38 days of EHM culture, tissues mounted on stretchers were carefully transferred to a custom-made Teflon holder, and placed into a 6-well culture plate filled with 8 mL of SFMM (each well). The pacing plate (C-Dish, IonOptix) was carefully positioned on top of the respective plates. Cells or tissues were then electrically paced at 1 or 3 Hz (studied in parallel for all experimental series) for 24 hours with biphasic 5 ms pulses at 20-25 V as previously described.[10,11] All electrical pacing was done at 37 °C, 5% $CO_2$. Pulses were delivered by a C-pace EM culture stimulator (IonOptix).

***Optogenetic stimulation of TC1133_CAG-Chrimson-YFP iPSC derived cardiomyocytes***

For optical pacing, a custom-made light source was positioned above a 6-well culture plate to deliver defined light pulses (118 µW/mm² intensity, 5 ms) at 1 or 3 Hz for 24 hours or 7 days. (**Figure S1**). All optical pacing was done at 37 °C, 5% $CO_2$. Media was the same as above for iPSC-CM and EHM. iPSC-CM optically paced for 7 days remained in their 6 well culture dish subsequent to differentiation. 7 day pacing was started on culture d35.

***Optical action potential recordings in iPSC-CM***

Action potentials in iPSC-CM were recorded as previously described.[12,13] Prior to the measurement, cells plated on glass coverslips were incubated with 0.1x VoltageFluor2.1Cl (Fluovolt, Thermo Scientific; 20 minutes loading). The coverslips were then transferred to a

37° C heated recording chamber filled with modified Tyrode's solution containing (in mmol/L): $CaCl_2$ 2, Glucose 10, HEPES 10, KCl 4, $MgCl_2$ 1, NaCl 140; pH = 7.35 with NaOH. Cells were then field stimulated at 1 Hz using an external pacing device (MyoPacer Field Stimulator, IonOptix), delivering 3-5 ms bipolar electrical pulses, set with a voltage 25% above stimulation threshold. Optical action potential signals from isolated and intact iPSC-CM were acquired using an inverted epifluorescence microscope ($\lambda_{ex}$ = 470 nm, $\lambda_{em}$ = 535 nm), optimized for high-speed signal capture with a photomultiplier (Cairn Research), digitized (Axon Digidata 1550B, Axon Instruments), and acquired with pClamp softare (Molecular Devices). *A posteriori* analysis was performed by ensemble averaging of 3 stable action potentials from a single cell.

***Voltage-clamp measurements of inward-rectifier $K^+$ currents***

Inward rectifier $K^+$ currents were recorded using whole-cell ruptured patch configuration. Membrane currents were acquired and analyzed using Axopatch 200B amplifier and pClamp-Software (V 10.7 Molecular Devices). Myocytes were continuously perfused with bath solution containing (in mmol/L): NaCl 120, KCl 20, $MgCl_2$ 1, $CaCl_2$ 2, glucose 10, HEPES 10, pH=7.4 at 22-24 °C. Borosilicate glass microelectrodes had tip resistances of 3-5 MΩ when filled with pipette solution (in mmol/L): K-aspartate 100, NaCl 10, KCl 40, Mg-ATP 5, EGTA 2, GTP-Tris 0.1, HEPES 10, pH 7.4). Seal resistances were 4–8 GΩ. Series resistance and cell capacitance were compensated. Inward rectifier potassium currents were measured by applying a ramp pulse from −100 to +40 mV (0.5 Hz). Agonist-inducible $I_{K,ACh}$ was stimulated with carbachol (CCh, 2 µmol/L). Agonist-independent constitutive $I_{K,ACh}$ activity was determined using the selective $I_{K,ACh}$ blocker tertiapin (TTP, 100 nmol/L) in a bath solution containing bovine serum albumin (0.1 g/L). $I_{K1}$ was identified as $Ba^{2+}$ (1 mmol/L)-sensitive current as previously described.[14–16]

***Automated voltage-clamp measurements of $I_{Ca,L}$ and $I_{Na}$***

All $I_{Ca,L}$ and $I_{Na}$ experiments were performed using the automated patch-clamp device, SyncroPatch 384 (Nanion Technologies GmbH) at room temperature.[17] Negative pressure (150-250 mbar) application attained whole-cell configuration. PatchControl 384 (Nanion Technologies GmbH) software was used for data acquisition. Thin borosilicate glass384-well planar chips (1xS-type NPC-384T) were used for all experiments. Recordings were excluded if

they showed a seal resistance of <250 MΩ, a peak current of <50 pA, or an $R_{series}$ of >20 MΩ (at 10 mV). At the start of an experiment, chips were loaded with 30 µL of a solution containing (in mmol/L): HEPES 10, NaCl 140, KCl 4, glucose 5. Following application of the desired pipette solution to generate electrical contact between external and internal sides of the chip, 20 µL of cell suspension was added to each well. Directly following this, 40 µL of a solution containing (in mmol/L): HEPES 10, NaCl 130, KCl 4, $CaCl_2$ 10, $MgCl_2$ 1, 5 glucose 5 was temporarily added to aid giga seal formation. 40 µL was then removed from each well and replaced with bath solution (detailed below).

*L-type calcium currents* ($I_{Ca,L}$) were measured at 0.5 Hz using a voltage-step protocol with a holding potential of -80 mV and a 100 ms ramp pulse to -40 mV followed by a 100 ms test-pulse to +10 mV at 22-24 °C. Pipette solution contained (in mmol/L): EGTA 10, HEPES 10, CsCl 10, NaCl 10, CsF 110, pH 7.2 (with CsOH). Bath solution contained (in mmol/L): HEPES 10, NaCl 140, glucose 5, KCl 4, $CaCl_2$ 2, $MgCl_2$ 1, pH 7.4 (with KOH). Offline analysis was performed with DataControl 384 software (Nanion Technologies GmbH). The integrated $I_{Ca,L}$ represents an estimate of $Ca^{2+}$ entering the cell, calculated per liter total cell volume, using a capacitance to volume relationship of 4.57 pF/pL.[18]

*Peak sodium currents* ($I_{Na}$) recordings were performed at 0.5 Hz using a voltage step protocol with a holding potential of -100 and a 30 ms test pulse to -20 mV followed by a step to -80 mV for 50 ms at 22-24 °C. Pipette solution contained (in mmol/L): EGTA 10, HEPES 10, KCl 10, NaCl 10, KF 110, pH 7.2 (with KOH). Bath solution contained (in mmol/L): HEPES 10, NaCl 140, glucose 5, KCl 4, $CaCl_2$ 2, $MgCl_2$ 1, pH 7.4 (with KOH). Offline analysis was performed with DataControl 384 software (Nanion Technologies GmbH).

*Basal Inward rectifier currents* ($I_{K1}$) were measured using automated patch clamp in cells optically paced for 7 days. Recordings were performed at 0.5 Hz by applying a ramp pulse from −100 to +40 mV at 22-24 °C. Pipette solution contained (in mmol/L): EGTA 10, HEPES 10, KCl 10, NaCl 10, KF 110, pH 7.2 (with KOH). Bath solution contained (in mmol/L): HEPES 10, NaCl 80, NMDG 60, glucose 5, KCl 20, $CaCl_2$ 2,$MgCl_2$ 1, pH 7.4 (with KOH). $I_{K1}$ was identified as $Ba^{2+}$ (1 mmol/L)-sensitive current. Offline analysis was performed with DataControl 384 software (Nanion Technologies GmbH).

***Sharp microelectrode measurements***

Action potentials in EHM were recorded as previously described.[19] Electrophysiological measurements were performed in a custom-built recording chamber perfused with heated (37 °C) and carbonated (5% $CO_2$) bath solution containing (in mmol/L): NaCl 126.7, KCl 5.4, $MgCl_2$ 1.1, $CaCl_2$ 1.8, $NaHPO_4$ 0.42, $NaHCO_3$ 22, glucose 5.5, pH=7.45. Borosilicate glass capillaries with an electrical resistance of 25-35 MΩ were pulled using a horizontal pipette puller and resulting pipettes were backfilled with 3 mol/L KCl solution. Signal was recorded and analyzed using a Sec-05-X amplifier (npi Electronic) in voltage follower mode and LabChart Pro 7 software (ADInstruments).

Tissue was electrically stimulated using a custom-made coaxial electrode (FHC) with a monophasic pulse of 1 ms and 2x threshold intensity. After successful tissue impalement and steady state signal acquisition, triggered action potentials were then recorded whilst stimulating the tissue at 1 Hz. Analysis was based on 10 consecutive and stable action potential recordings in the same cell.

***Contractile force analysis***

Separate aEHM were prepared in an alternative engineering format to determine the effects of optical tachypacing on contractility over an extended period of time *in situ* as previously described.[9] (construct here termed bioartificial cardiac tissue [BCT]). aEHM were placed into custom-made culture vessels with integrated force measurement capabilities as previously described.[20] (construct here termed bioartificial cardiac tissue [BCT]). Force of contraction was first measured at 1 Hz in all tissues to measure the baseline force (day 0), then tissues were optically paced at 1 Hz (n=3 tissues) or 3 Hz (n=6 tissues) for 7 days. Once every 24 hours, contractile forces of both groups were measured at 1 Hz. On day 7, all tissues received 1 Hz optical pacing for an additional 7 days to test for contractile force recovery (**Figure S15**). All tissue culture and measurements were conducted at 37 °C, 5% $CO_2$. Analysis via the averaging of 30 contractile signals during 1 Hz measurement every 24 hours was performed using a custom Python-based analysis software.

***FACs analysis***

For flow cytometric analysis of atrial iPSC-CM, the cells were dissociated with Trypisn/EDTA (PAN-Biotech) into single cells, fixed with 4% FA at RT for 10 minutes, permeabilized with 90% ice-cold methanol for 15 min followed by a blocking step with 3% BSA and 5% goat serum for 20 min. Cells were then incubated with antibodies against the atrial (MLC2a) and the ventricular (MLC2v) isoform of myosin light chain for 1h at RT. Cells were then incubated with secondary antibody in 3% BSA in PBS at RT for 30 min. Antibodies are listed below in **Supplemental Table 1**. Cells were subsequently analyzed using the BD Accuri$^{TM}$ C6 plus system flow cytometer (BD Biosciences). Isotype control for each primary antibody was used as negative control. 30,000 events were acquired and at least 20,000 events were analyzed per sample.

**Supplemental Table 1. Antibodies used in FACs analysis**

| Antibody | Concentration | Company | Identifier |
|---|---|---|---|
| MLC2a | 1:200 | Synaptic Systems | 311 011 |
| Mouse IgG2b | 1:200 | Dako | X0944 |
| MLC2v | 1:100 | Proteintech | 10906-1-AP |
| Rabbit IgG | 1:1600 | Abcam | ab37415 |
| Donkey Anti Mouse IgG - Cy3 | 1:1000 | JIR | 715-165-150 |
| Goat Anti Rabbit IgG - Cy2 | 1:1000 | JIR | 111-225-144 |

***Gene-expression analysis***

Total RNA was isolated from iPSC-CM EHM and native human tissue samples using TRIzol (Thermo Fisher Scientific, Waltham, MA, USA, #15596018) phenol-chloroform extraction. Remaining genomic DNA was removed using RNase-free DNase I (Thermo Fisher Scientific, Waltham, MA, USA, #EN0521). 1 µg DNAse-treated RNA was transcribed into complementary DNA (cDNA) using RevertAid First Strand cDNA synthesis kit (Thermo Fisher Scientific, Waltham, MA, USA, #K1622) and random hexamer primers. Quantitative real-time reverse transcriptase polymerase chain reaction (qPCR) was performed using SsoAdvancedTM Universal SYBR Green supermix (Bio-Rad, California, USA, #1725271) and 25 ng cDNA was used as template per reaction. Fluorescence detection was started with 10 min incubation at 95 °C, followed by 40 cycles at 95 °C for 15 seconds and 60 °C for 1 min. After 40 cycles, melt curve analysis was performed to ensure amplicon specificity. For relative quantification, the expression levels of all target genes were normalized to the housekeeping gene GAPDH. Relative gene expression levels were calculated using ΔΔCq method by subtracting the ΔCq of samples paced with 3 Hz from the average of ΔCq of samples paced with 1 Hz. Primer sequences are listed in **Supplemental Table 2.**

**Supplemental Table 2. Primer sequences (5´ to 3´) for qPCR obtained from PrimerBank (https://pga.mgh.harvard.edu/primerbank/).**

| Gene | Forward primer | Reverse primer |
|---|---|---|
| *GAPDH* | GGAAGGTGAAGGTCGGAGTCA | GTCATTGATGGCAACAATATCCACT |
| *CACNA1C* | AATCGCCTATGGACTCCTCTT | GCGCCTTCACATCAAATCCG |
| *KCNJ2* | GTGCGAACCAACCGCTACA | CCAGCGAATGTCCACACAC |
| *KCNJ3* | TCGGCTATGGCTACCGATACA | GTGCTCGCTGAACATGAGG |
| *KCNJ5* | GCGCTTCAACTTGCTCGTC | GCCACTGAGGTTTTCAACACA |
| *ADORA1* | CCACAGACCTACTTCCACACC | TACCGGAGAGGGATCTTGACC |
| *CHRM2* | ACACCCTCTACACTGTGATTGG | GTCCGCTTGACTGGGTAGG |

## Supplemental Figures

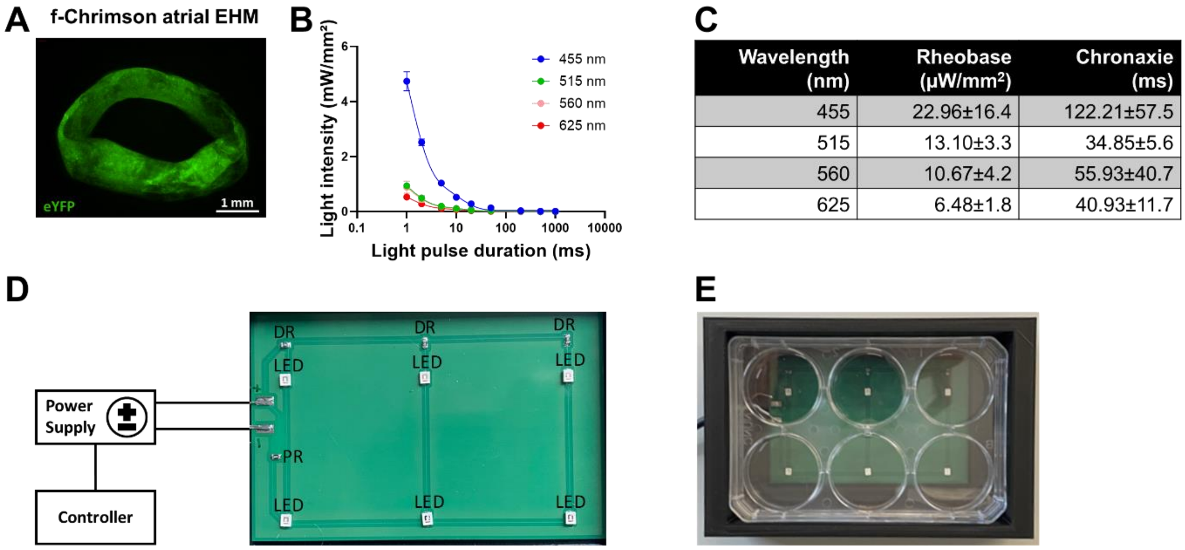


| Wavelength (nm) | Rheobase (µW/mm²) | Chronaxie (ms) |
|---|---|---|
| 455 | 22.96±16.4 | 122.21±57.5 |
| 515 | 13.10±3.3 | 34.85±5.6 |
| 560 | 10.67±4.2 | 55.93±40.7 |
| 625 | 6.48±1.8 | 40.93±11.7 |

**Figure S1. Optical pacing of atrial engineered human myocardium (aEHM) expressing the light-gated ion channel f-Chrimson. A,** Representative fluorescence image (YFP) of an aEHM expressing the light-gated ion channel f-Chrimson. **B,** Intensity-duration plot for different wavelengths which trigger contraction of aEHM. **C,** Rheobase and chronaxie. Rheobase is defined as minimal intensity of infinite duration required to trigger a contraction. Chronaxie is defined as minimum time required for an intensity double the strength of the rheobase to trigger contraction. **D,** Schematic circuit diagram of the custom-made LED-plate used for optical stimulation. DR, dropping resistor, PR, parallel resistor. **E,** Bottom view of LED-plate and housing covering a standard six-well culture plate.

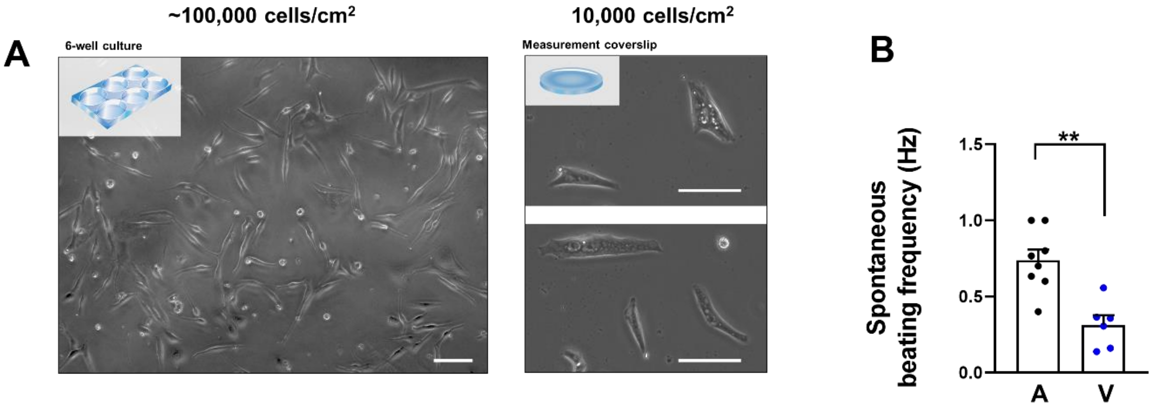


**Figure S2. Induced pluripotent stem cell derived cardiomyocyte (iPSC-CM) characteristics. A,** Photomicrographs of atrial iPSC-CM employed in this study in dense 6 well formats (left) prior to coverslip seeding, and sparsely plated monolayers on 10 mm glass coverslips (right) for live single cell measurements. Scale bar = 50 µm. **B**, Spontaneous beating frequency of atrial (A) and ventricular (V) iPSC-CM at day 28 analyzed optically at 37 °C. Atrial: n=8/1, Ventricular: n=6/1. Data are mean±SEM. **P<0.01 using Mann-Whitney-U test. n/N = number of iPSC-CM/differentiation.

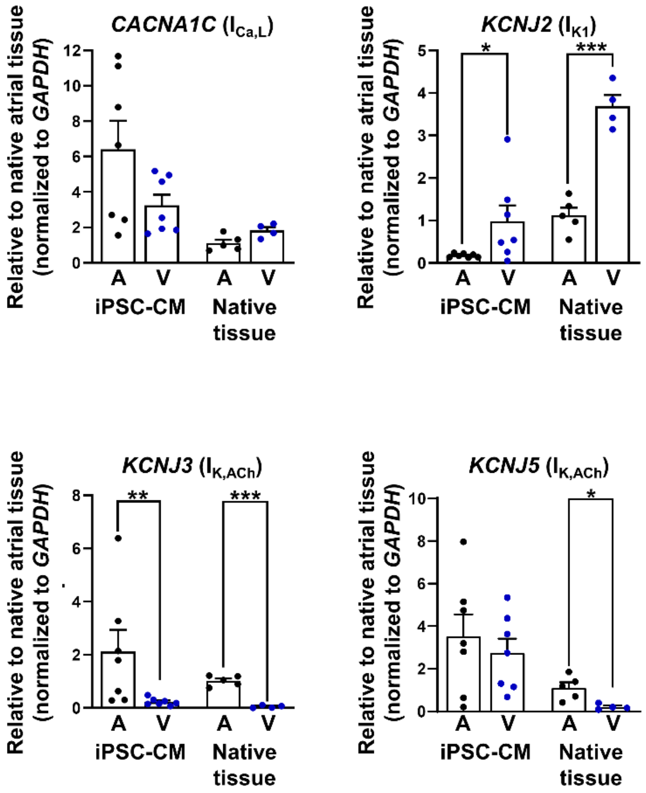


**Figure S3: mRNA expression levels of L-type $Ca^{2+}$ channels ($I_{Ca,L}$) and inward rectifier $K^+$ channels ($I_{K1}$, $I_{K,ACh}$) in atrial (iPSC-aCM, A) and ventricular (iPSC-vCM, V) iPSC-CM, and atrial and ventricular biopsies from patients.** iPSC-aCM: n=7 differentiations. iPSC-vCM: n=7 differentiations. Atrial biopsies: n=5 patients. Ventricular biopsies: n=4 patients. Data are mean±SEM normalized to atrial patient biopsies. *P<0.05, **P<0.01 and *** P<0.001 vs atrial using the Mann-Whitney U test.

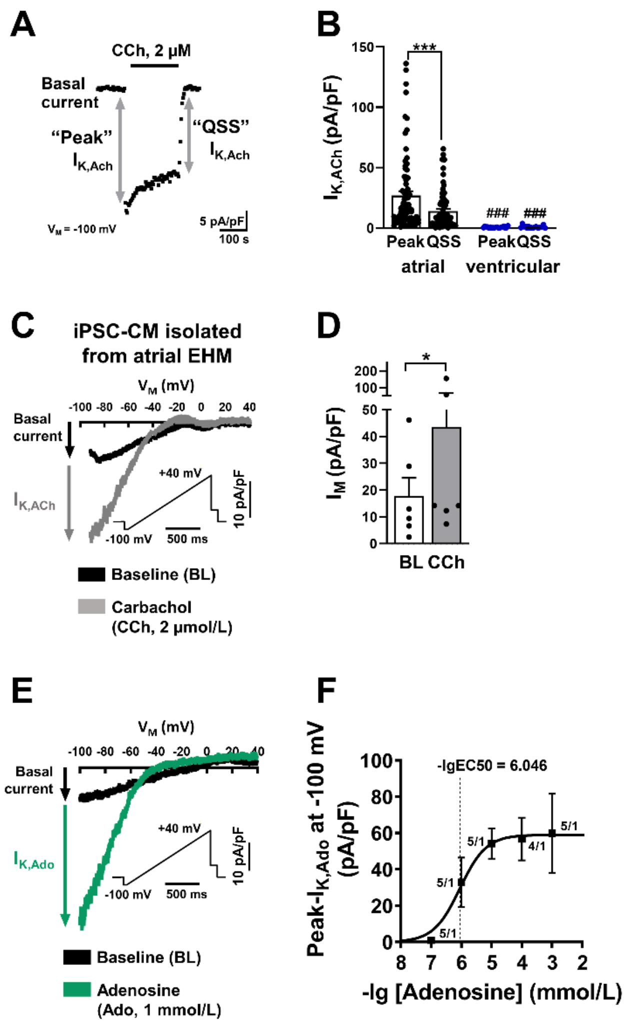


**Figure S4: Inward rectifier potassium currents in atrial induced pluripotent stem cell derived cardiomyocytes (iPSC-aCM). A,** Representative time course of inward rectifier current amplitude at -100 mV before, during and after application of the M-receptor agonist carbachol (CCh, 2 µmol/L). During activation, the initial increase (Peak) rapidly desensitized to a quasi steady-state (QSS). **B,** Peak $I_{K,ACh}$ and QSS in iPSC-aCM (n=77/6) and iPSC-vCM (n=31/4). **C,** Representative voltage-clamp recording of basal inward rectifier potassium current ($I_{K1}$) and CCh-activated $I_{K,ACh}$ in atrial iPSC-CM isolated from atrial EHM. **D,** Total inward rectifier currents (membrane current, $I_M$) at -100 mV (n=6/2). **E,** Representative voltage-clamp recording of basal inward rectifier potassium current ($I_{K1}$) and adenosine (Ado, 1 mmol/L)-activated $I_{K,Ado}$ in atrial iPSC-CM. **F,** Concentration-response curve for Ado-mediated activation of $I_{K,Ado}$ at -100 mV (n=4-5/1). Data are mean±SEM. $^{###}$*P*<0.001 vs. iPSC-aCM and ***$P$<0.001

using Welch's *t*-test and the Bonferroni multiple comparison test (**B**). *$P$<0.05 using paired Student's *t*-test (**D**). n/N = number of iPSC-CM/differentiation.

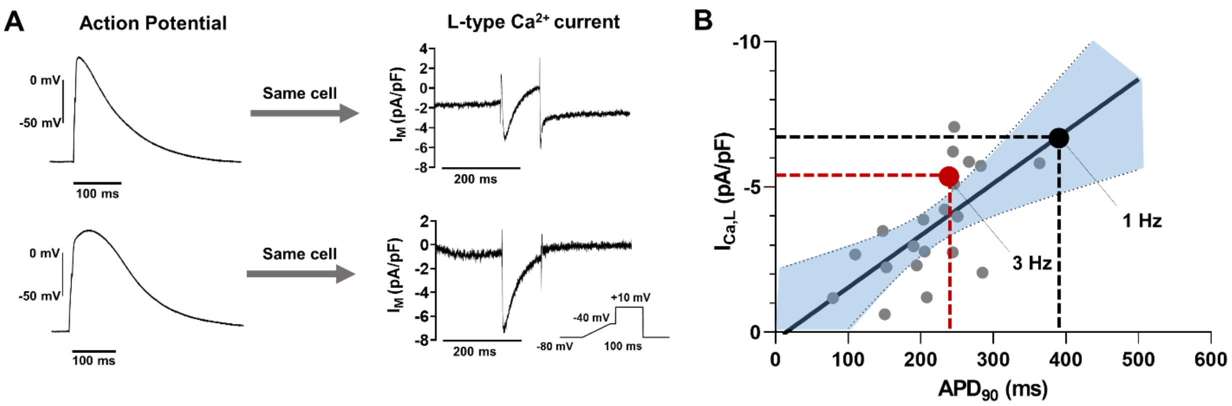


**Figure S5. Action potential (AP) duration dependence on L-type calcium current ($I_{Ca,L}$) in atrial induced pluripotent stem cell-derived cardiomyocytes (iPSC-aCM). A,** Representative AP traces elicited at 1 Hz from a single iPSC-aCM and the corresponding $I_{Ca,L}$ measured from the same cell following a switch of amplifier configuration and pipette solution using an automated patch clamp device (Syncropatch 384, Nanion Technologies GmbH). **B**, Linear regression of the AP duration at 90% repolarization ($APD_{90}$) and corresponding $I_{Ca,L}$ density in iPSC-aCM. The 95% confidence interval of the regression is shaded. The experimental averages obtained in this study following electrical pacing of iPSC-aCM for 24 hours at 1 Hz or 3 Hz (**Figure 3B, D**) are overlaid. This demonstrates the dependence of AP duration on $I_{Ca,L}$ density and represents a graphical means to estimate the contribution of $I_{Ca,L}$ to AP shortening in tachypaced conditions.

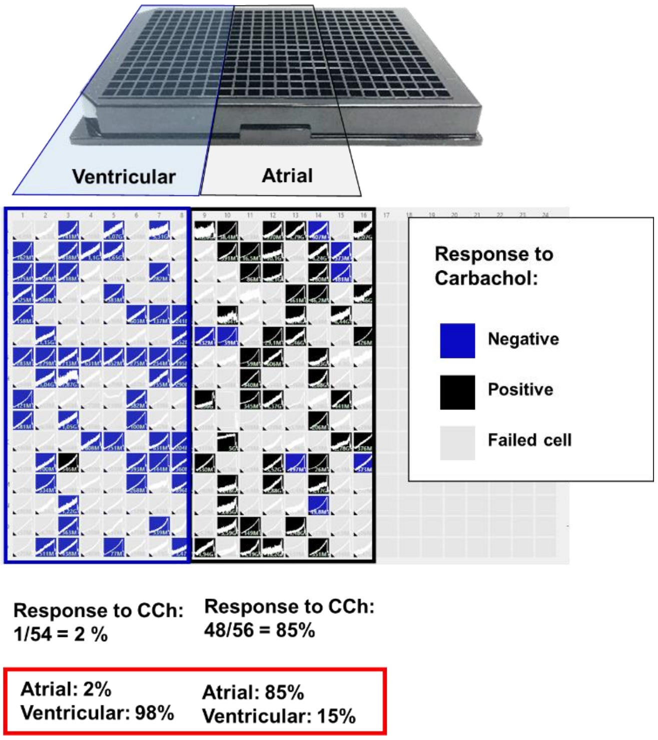


**Figure S6. High throughput functional screening for atrial induced pluripotent stem cell derived cardiomyocytes (iPSC-aCM) specificity by testing the basal inward rectifier potassium current ($I_{K1}$) at -100 mV in the presence of 2 µmol/L carbachol (CCh).** iPSC-aCM and Ventricular iPSC-CM (iPSC-vCM) distributed across a recording plate (top) for a high throughput automated patch clamp device (Syncropatch 384, Nanion Technologies GmbH). Upon measurement of inward rectifier currents as described in this study, a response to CCh can indicate the presence or absence of an atrial subtype. Global results from a single ventricular batch and a singular atrial batch are shown (bottom).

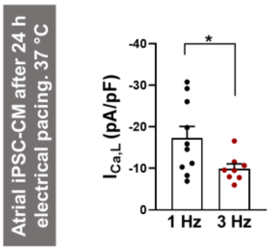


**Figure S7. 24 hour tachypacing-induced remodelling of L-type calcium current ($I_{Ca,L}$) in atrial induced pluripotent stem cell derived cardiomyocytes (iPSC-aCM).** Peak $I_{Ca,L}$ measured at 37 °C in iPSC-aCM after 24 h electrical pacing at 1 Hz or 3 Hz (1 Hz: n=10/2, 3 Hz: n=8/2). Data are mean±SEM.**P*<0.05 vs 1 Hz using unpaired Student's t test. n/N = number of iPSC-CM/differentiation.

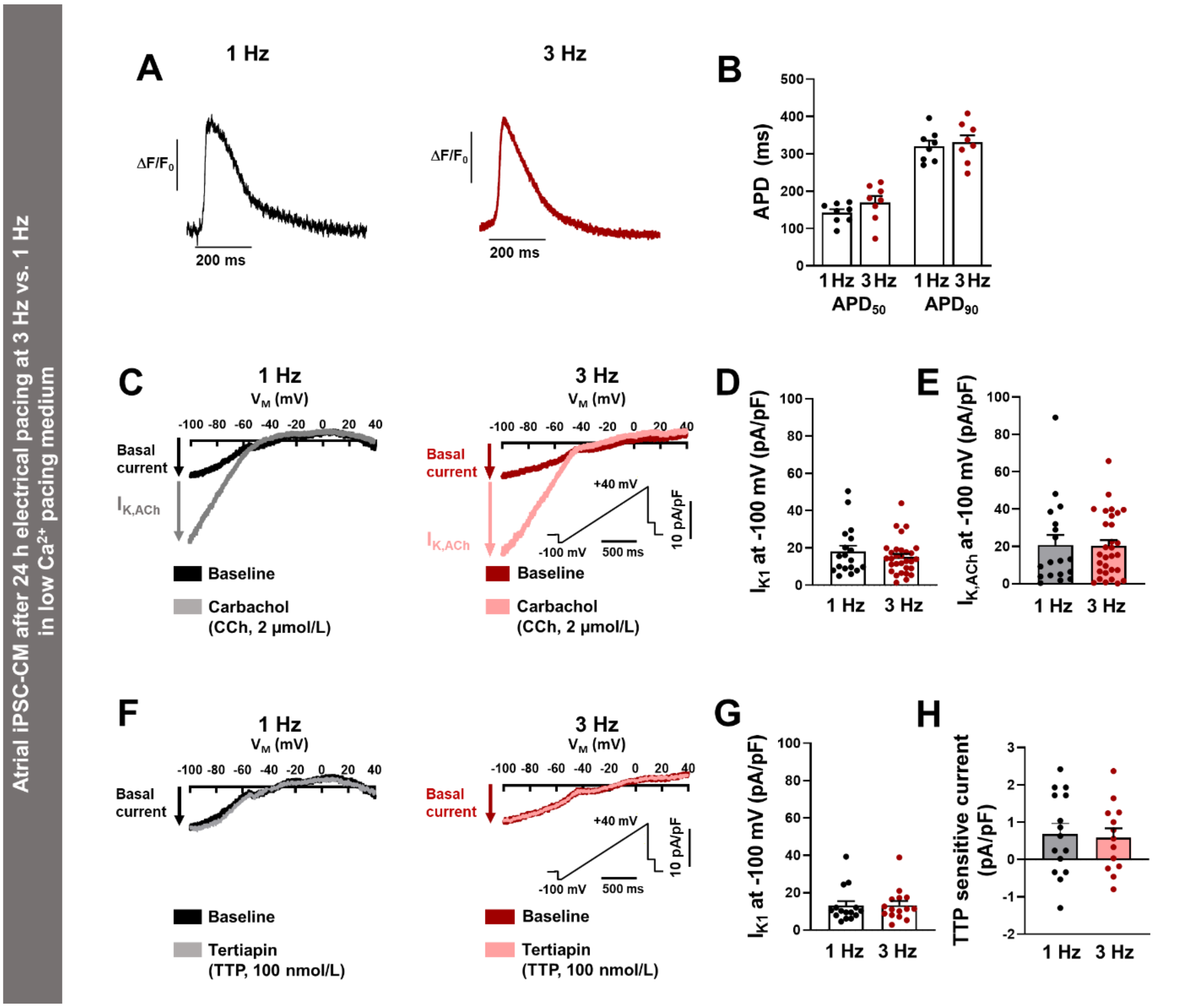


**Figure S8. Low-$Ca^{2+}$ pacing conditions prevented tachypacing-induced remodelling in atrial atrial induced pluripotent stem cell derived cardiomyocytes (iPSC-aCM).** Representative optical action potentials (AP) elicited at 1 Hz in single intact iPSC-aCM after 24 h electrical pacing in low-$Ca^{2+}$ pacing medium (0.42 mmol/L $Ca^{2+}$, [normal pacing occurs in 1.79 mmol/L $Ca^{2+}$]) at 1 Hz (left) or 3 Hz (right). **B,** AP duration at 50% and 90% repolarization ($APD_{50}$, $APD_{90}$, 1 Hz: n=8/2, 3 Hz: n=8/2). **C**, Representative voltage-clamp recordings of basal inward-rectifier $K^+$ current ($I_{K1}$) current in isolated iPSC-aCM after 24 h electrical pacing in low-$Ca^{2+}$ pacing medium at 1 Hz (left) or 3 Hz (right) before (baseline) and after application of the M-receptor agonist carbachol (CCh, 2 µmol/L), revealing the acetylcholine activated inward-rectifier $K^+$ current ($I_{K,ACh}$). **D**, Peak $I_{K1}$ measured at -100 mV. **E**, Peak $I_{K,ACh}$ measured at -100 mV (**D,E**: 1 Hz: n=18/2, 3 Hz n=30/2). **F**, Representative voltage-clamp recordings of $I_{K1}$ in isolated iPSC-aCM after 24 h electrical pacing in low-$Ca^{2+}$ pacing medium at 1 Hz (left) or 3 Hz (right) before (baseline) and after application of selective $I_{K,ACh}$ blocker tertiapin (TTP, 100 nmol/L). **G**, Peak $I_{K1}$ measured at -100 mV. **H**, Change in basal current at -100 mV following TTP application,

defined as agonist independent constitutive $I_{K,ACh}$ ($I_{K,ACh,c}$; **G, H**: 1 Hz: n=15/2, 3 Hz n=13/2). Data are mean±SEM. n/N = number of iPSC-CM/differentiation.

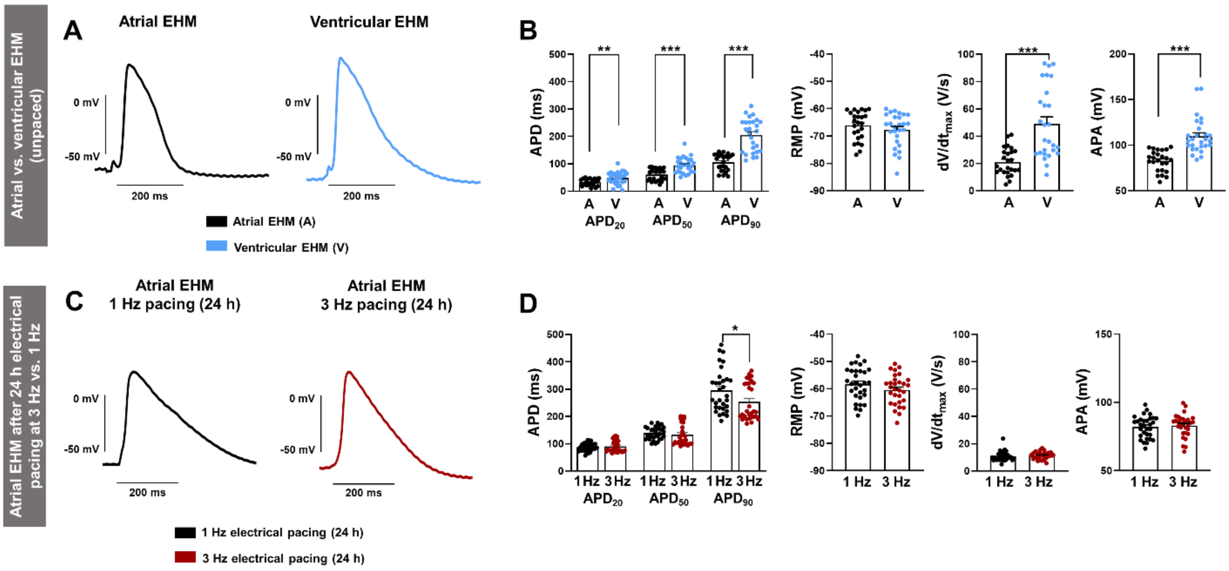


**Figure S9. Electrical remodelling of atrial engineered human myocardium (aEHM) induced by 24-hour electrical tachypacing. A,** Representative action potentials (AP) elicited at 1 Hz in aEHM (left) and ventricular (vEHM, right) EHM. **B**, AP duration at 20%, 50% and 90% repolarization ($APD_{20}$, $APD_{50}$ and $APD_{90}$, left), resting membrane potential (RMP, middle left), upstroke velocity ($dV/dt_{max}$, middle right), AP amplitude (APA, right; atrial: n=57/11, ventricular: n=27/6). **C,** Representative action potential (AP) elicited at 1 Hz in aEHM after 24 h electrical pacing at 1 Hz (left) or 3 Hz (right). **D**, AP duration at 20%, 50% and 90% repolarization ($APD_{20}$, $APD_{50}$ and $APD_{90}$, left), resting membrane potential (RMP, middle left), upstroke velocity ($dV/dt_{max}$, middle right), AP amplitude (APA, right; 1 Hz: n=30/8, 3 Hz: n=30/8). Data are mean±SEM. ***P*<0.01, ****P*<0.001 vs aEHM or **P*<0.05 vs 1 Hz using unpaired Student's t test. n/N = number of recordings/EHM.

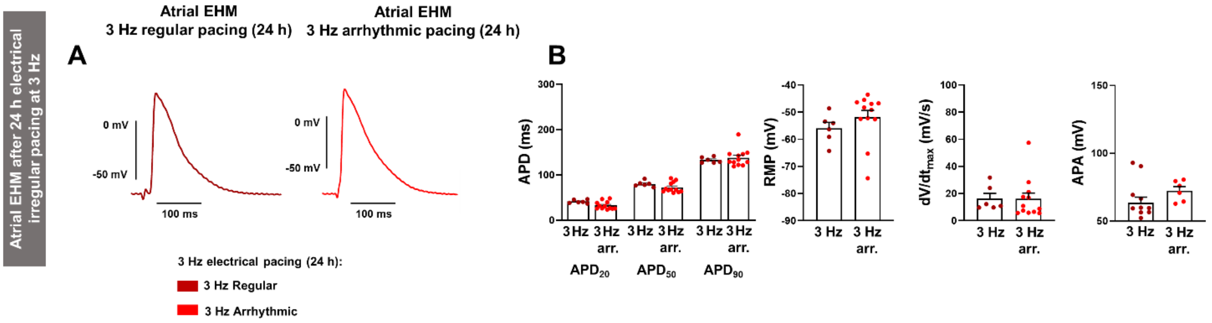


**Figure S10. Impact of arrhythmic pacing (arr.) on electrical remodelling of atrial engineered human myocardium (aEHM) induced by 24-hour electrical tachypacing. A,** Representative action potential (AP) elicited at 1 Hz in aEHM after 24 h electrical pacing at 3 Hz (left) or at a 3 Hz arrhythmic frequency with 50% variability (right). **B**, AP duration at 20%, 50% and 90% repolarization ($APD_{20}$, $APD_{50}$ and $APD_{90,}$ left), resting membrane potential (RMP, middle left), upstroke velocity ($dV/dt_{max}$, middle right), AP amplitude (APA, right; 3 Hz: n=6/3, 3 Hz: n=12/4). Data are mean±SEM. n/N = number of recordings/EHM.

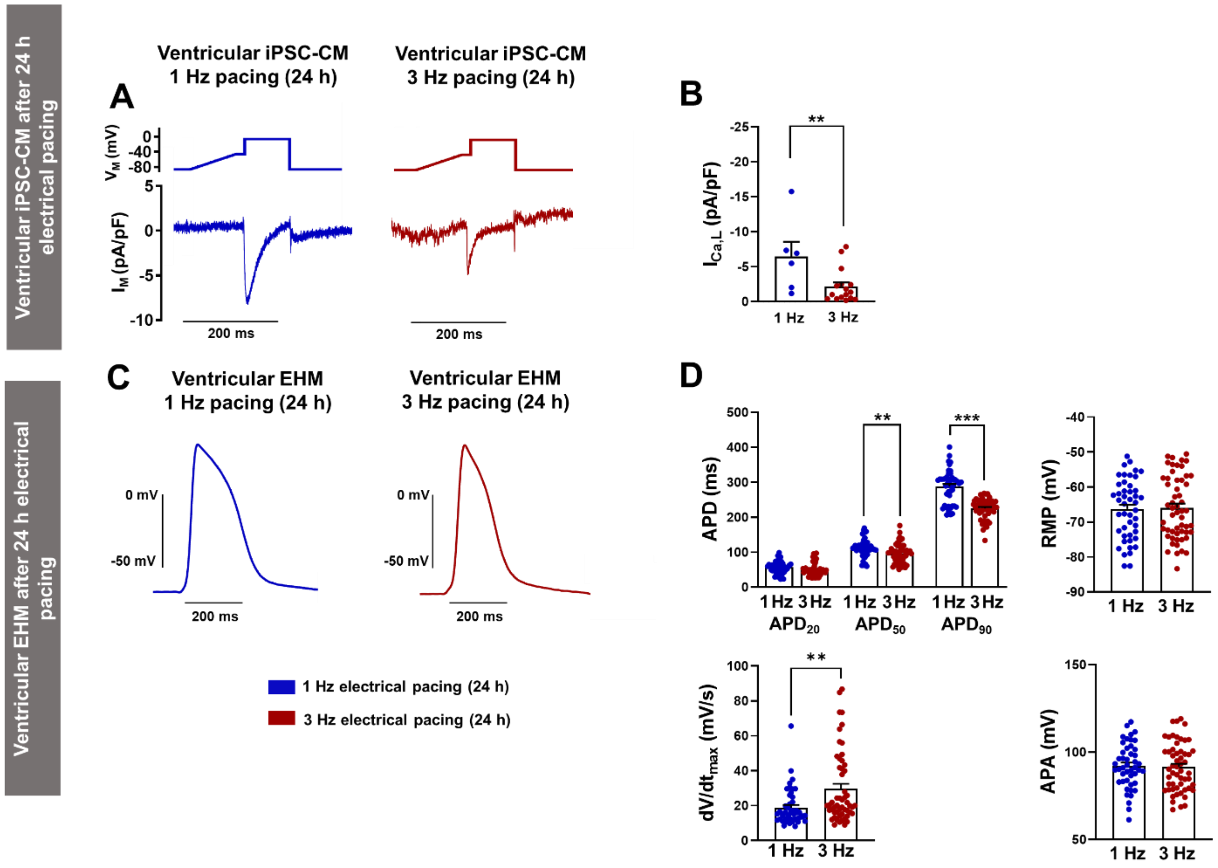


**Figure S11. 24 hour tachypacing-induced remodelling of cellular electrophysiology in ventricular induced pluripotent stem cell derived cardiomyocytes (iPSC-vCM) and ventricular engineered human myocardium (vEHM). A,** Voltage-clamp protocol (0.5 Hz, top) and representative membrane current ($I_M$) trace (bottom) of L-type $Ca^{2+}$ current ($I_{Ca,L}$) in iPSC-vCM after 24 h electrical pacing at 1 Hz (left) or 3 Hz (right). **B**, Peak $I_{Ca,L}$ (1 Hz: n=6/1, 3 Hz: n=16/1). **C**, Representative AP elicited at 1 Hz in vEHM after 24 h electrical pacing at 1 Hz (left) or 3 Hz (right). **D**, AP duration at 20%, 50% and 90% repolarization ($APD_{20}$, $APD_{50}$ and $APD_{90}$, top left), resting membrane potential (RMP, top right), upstroke velocity ($dV/dt_{max}$, bottom left), AP amplitude (APA, bottom right; 1 Hz: n=46/15, 3 Hz: n=55/20). Data are mean±SEM. ***P*<0.01, ***P<0.001 vs 1 Hz using unpaired Student's t test. n/N = number of iPSC-CM/differentiation or number of recordings/EHM.

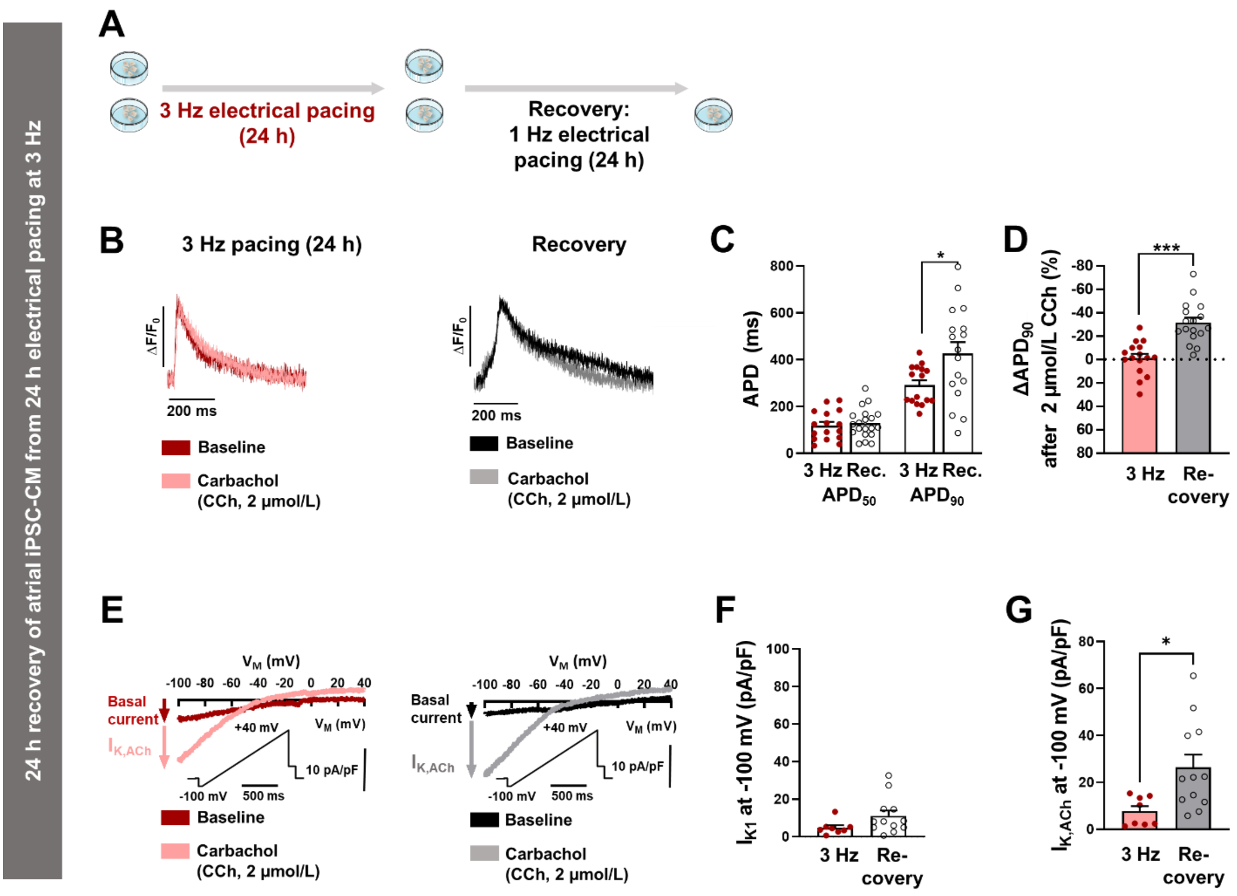


**Figure S12. 24 hour recovery of atrial induced pluripotent stem cell derived cardiomyocytes (iPSC-aCM) following 3 Hz electrical pacing. A,** Schematic of the recovery protocol. After 24 h 3 Hz tachypacing, iPSC-aCM were subjected to normofrequent pacing at 1 Hz for 24 h. **B**, Representative optical action potentials (AP) elicited at 1 Hz in single intact iPSC-aCM after 24 h electrical pacing at 3 Hz (left) or a further 24 h at 1 Hz (recovery, right) before (baseline) and after application of the M-receptor agonist carbachol (CCh, 2 µmol/L). **C**, AP duration at 50% and 90% repolarization. **D**, Percentage change of AP duration at 90% repolarization ($APD_{90}$) following CCh application (**C, D**: 3 Hz: 16/3, Recovery: n=17/3). **E**, Representative voltage-clamp recordings of basal inward-rectifier $K^+$ current ($I_{K1}$) current in isolated iPSC-aCM after 24 h electrical pacing at 3 Hz (left) or a further 24 h at 1 Hz (recovery, right) before (baseline) and after CCh application, revealing the acetylcholine activated inward-rectifier $K^+$ current ($I_{K,ACh}$). **F**, Peak $I_{K1}$ measured at -100 mV. **G**, Peak $I_{K,ACh}$ measured at -100 mV (**F,G**: 3 Hz: n=8/1, Recovery: n=12/1). Data are mean±SEM *P<0.05, ***P<0.001 using unpaired Student's *t*-test and Welch's *t*-test (**G**). n/N = number of iPSC-CM/differentiation.

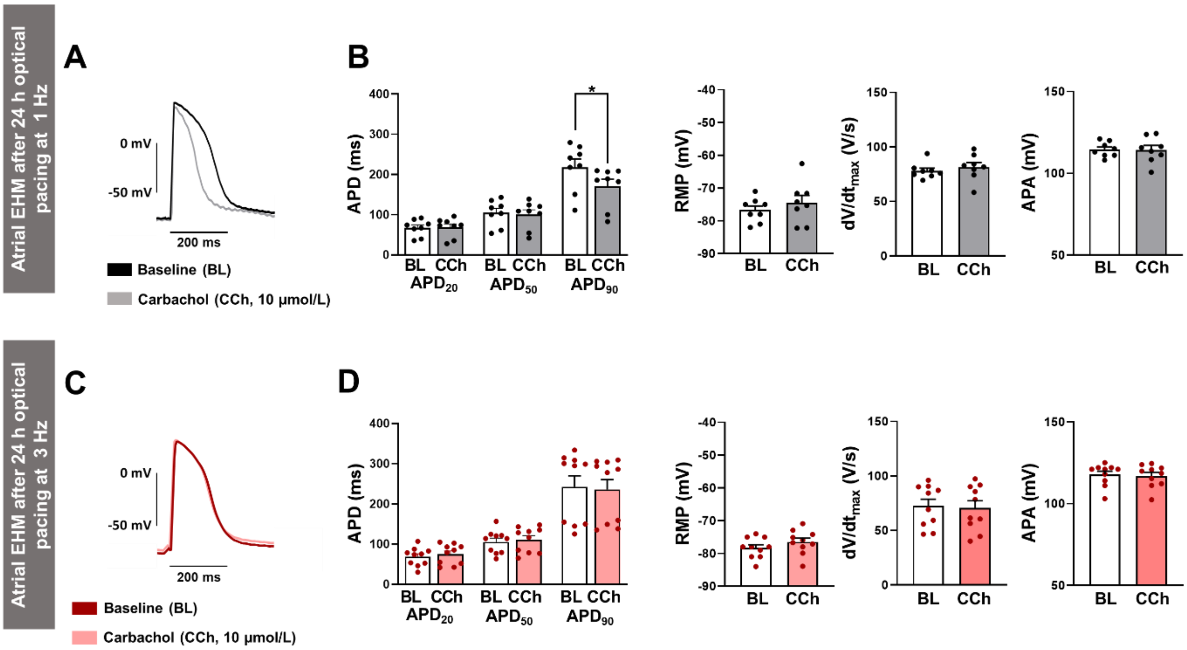


**Figure S13. 24 hour optical tachypacing-induced electrical remodelling of atrial engineered human myocardium (aEHM). A,** Representative action potentials (AP) elicited at 1 Hz in aEHM subjected to 1 Hz 24-hour optical pacing before (baseline, BL) and after application of the M-receptor agonist carbachol (CCh, 10 µmol/L). (Replotted from Figure 4H). **B**, AP duration at 20%, 50% and 90% repolarization ($APD_{20}$, $APD_{50}$ and $APD_{90}$, left), resting membrane potential (RMP, middle left), upstroke velocity ($dV/dt_{max}$, middle right), AP amplitude (APA, right) at BL and after CCh application (1 Hz: n=8/5). **C**, Representative AP elicited at 1 Hz in aEHM subjected to 3 Hz 24-hour optical pacing before (baseline, BL) and after application of CCh. (Replotted from Figure 4H). **D**, $APD_{20}$, $APD_{50}$ and $APD_{90}$, (left), RMP (middle left), $dV/dt_{max}$, (middle right), APA (right) at BL and after CCh application (3 Hz: n=10/5). Data are mean±SEM.**P*<0.05 vs BL using paired Student's t test. n/N = number of recordings/EHM.

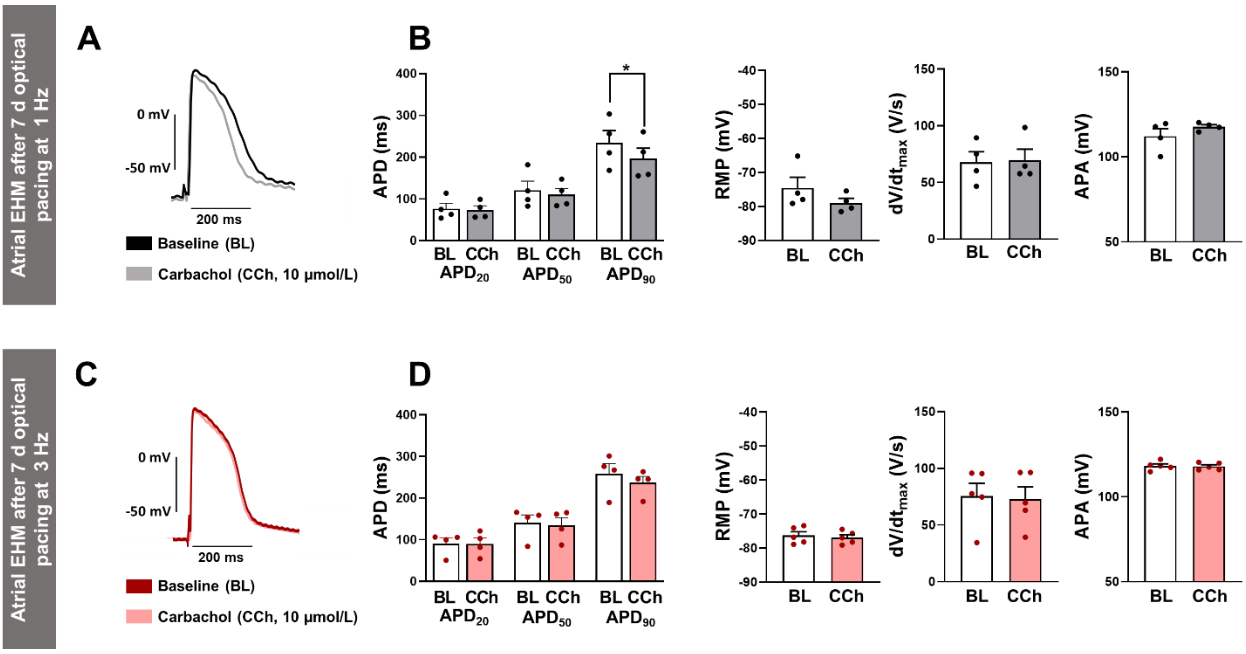


**Figure S14. 7 day optical tachypacing-induced electrical remodelling of atrial engineered human myocardium (aEHM). A,** Representative action potentials (AP) elicited at 1 Hz in aEHM subjected to 1 Hz 7 day optical pacing before (baseline, BL) and after application of the M-receptor agonist carbachol (CCh, 10 µmol/L). **B**, AP duration at 20%, 50% and 90% repolarization ($APD_{20}$, $APD_{50}$ and $APD_{90}$, left), resting membrane potential (RMP, middle left), upstroke velocity ($dV/dt_{max}$, middle right), AP amplitude (APA, right) at BL and after CCh application (1 Hz: n=4/4). **C**, Representative AP elicited at 1 Hz in aEHM subjected to 3 Hz 24-hour optical pacing before (baseline, BL) and after application of CCh. (Replotted from Figure 4H). **D**, $APD_{20}$, $APD_{50}$ and $APD_{90}$, (left), RMP (middle left), $dV/dt_{max}$, (middle right), APA (right) at BL and after CCh application (3 Hz: n=5/4). Data are mean±SEM.**P*<0.05 vs BL using paired Student's t test. n/N = number of recordings/EHM.

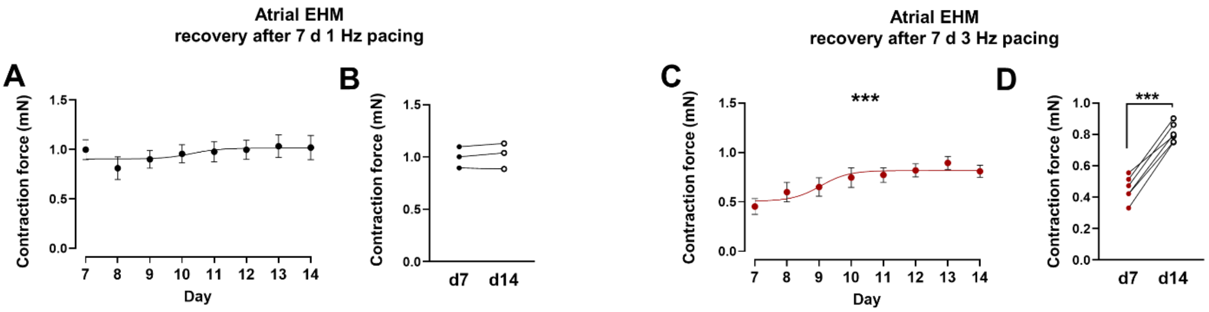


**Figure S15. Recovery of 7 day optical tachypacing-induced contractile dysfunction in atrial engineered human myocardium (aEHM). A**, Time course of force of contraction of aEHM optically paced for 7+7 days at 1 Hz (n=3). **B**, Change in contractile force between day 7 (d7) and d14 after 7+7 day optical pacing at 1 Hz. Single point data extracted from (**A**). **C**, Time course of contractile function of aEHM after optical pacing for 7 days at 3 Hz, now exposed to 7 days of 1 Hz continuous pacing (n=6). **D**, Change in contractile force between day 7 (d7) and d14 of optical pacing at 1 Hz following 7 days of 3 Hz pacing. Single point data extracted from (**C**). Data are mean±SEM. ****P*<0.001 vs d7 using paired Student's t test (**E, G**) or vs 1 Hz with an extra sum of squares F test to compare fitted sigmoidal curves (**A,C**). n = number of EHM.

## Supplemental Videos

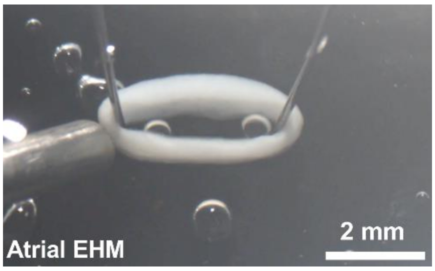


**Supplementary video 1. (screenshot) aEHM spontaneously beating in bath solution prior to an experimental sharp electrode measurement.**

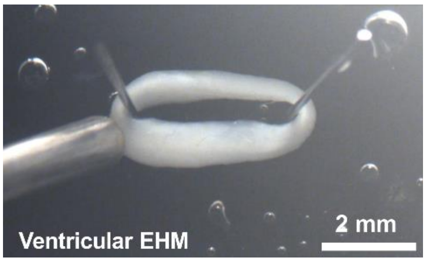


**Supplementary video 2. (screenshot) Ventricular EHM spontaneously beating in bath solution prior to an experimental sharp electrode measurement.**

## Supplemental References

1. Baghbaderani BA, Tian X, Neo BH, Burkall A, Dimezzo T, Sierra G, Zeng X, Warren K, Kovarcik DP, Fellner T, Rao MS. CGMP-manufactured human induced pluripotent stem cells are available for pre-clinical and clinical applications. *Stem Cell Reports* 2015;**5**:647–659.

2. Cyganek L, Tiburcy M, Sekeres K, Gerstenberg K, Bohnenberger H, Lenz C, Henze S, Stauske M, Salinas G, Zimmermann WH, Hasenfuss G, Guan K. Deep phenotyping of human induced pluripotent stem cell-derived atrial and ventricular cardiomyocytes. *JCI insight* 2018;**3**.

3. Burridge PW, Matsa E, Shukla P, Lin ZC, Churko JM, Ebert AD, Lan F, Diecke S, Huber B, Mordwinkin NM, Plews JR, Abilez OJ, Cui B, Gold JD, Wu JC. Chemically defned generation of human cardiomyocytes. *Nat Methods* 2014;**11**:855–860.

4. Lian X, Zhang J, Azarin SM, Zhu K, Hazeltine LB, Bao X, Hsiao C, Kamp TJ, Palecek SP. Directed cardiomyocyte differentiation from human pluripotent stem cells by modulating Wnt/β-catenin signaling under fully defined conditions. *Nat Protoc* 2013;**8**:162–175.

5. Lee JH, Protze SI, Laksman Z, Backx PH, Keller GM. Human Pluripotent Stem Cell-Derived Atrial and Ventricular Cardiomyocytes Develop from Distinct Mesoderm Populations. *Cell Stem Cell* 2017;**21**:179-194.e4.

6. Tohyama S, Hattori F, Sano M, Hishiki T, Nagahata Y, Matsuura T, Hashimoto H, Suzuki T, Yamashita H, Satoh Y, Egashira T, Seki T, Muraoka N, Yamakawa H, Ohgino Y, Tanaka T, Yoichi M, Yuasa S, Murata M, Suematsu M, Fukuda K. Distinct metabolic flow enables large-scale purification of mouse and human pluripotent stem cell-derived cardiomyocytes. *Cell Stem Cell* 2013;**12**:127–137.

7. Tiburcy M, Hudson JE, Balfanz P, Schlick S, Meyer T, Liao MLC, Levent E, Raad F, Zeidler S, Wingender E, Riegler J, Wang M, Gold JD, Kehat I, Wettwer E, Ravens U, Dierickx P, Laake LW Van, Goumans MJ, Khadjeh S, Toischer K, Hasenfuss G, Couture LA, Unger A, Linke WA, Araki T, Neel B, Keller G, Gepstein L, Wu JC, Zimmermann WH. Defined engineered human myocardium with advanced maturation for applications in heart failure modeling and repair. *Circulation* 2017;**135**:1832–1847.

8. Tiburcy M, Meyer T, Liaw NY, Zimmermann WH. Generation of Engineered Human Myocardium in a Multi-well Format. *STAR Protoc* 2020;**1**.

9. Kensah G, Roa Lara A, Dahlmann J, Zweigerdt R, Schwanke K, Hegermann J, Skvorc D, Gawol A, Azizian A, Wagner S, Maier LS, Krause A, Dräger G, Ochs M, Haverich A, Gruh I, Martin U. Murine and human pluripotent stem cell-derived cardiac bodies form contractile myocardial tissue in vitro. *Eur Heart J* 2013;**34**:1134–1146.

10. Makary S, Voigt N, Maguy A, Wakili R, Nishida K, Harada M, Dobrev D, Nattel S. Differential protein kinase c isoform regulation and increased constitutive activity of acetylcholine-regulated potassium channels in atrial remodeling. *Circ Res* 2011;**109**:1031–1043.

11. Qi XY, Yeh YH, Xiao L, Burstein B, Maguy A, Chartier D, Villeneuve LR, Brundel BJJM, Dobrev D, Nattel S. Cellular signaling underlying atrial tachycardia remodeling of L-type calcium current. *Circ Res* 2008;**103**:845–854.

12. Seibertz F, Reynolds M, Voigt N. Single-cell optical action potential measurement in human induced pluripotent stem cell-derived cardiomyocytes. *J Vis Exp* 2020;**2020**:e61890.

13. Peper J, Kownatzki-Danger D, Weninger G, Seibertz F, Pronto JRD, Sutanto H, Pacheu-Grau D, Hindmarsh R, Brandenburg S, Kohl T, Hasenfuss G, Gotthardt M, Rog-Zielinska EA, Wollnik B, Rehling P, Urlaub H, Wegener J, Heijman J, Voigt N, Cyganek L, Lenz C, Lehnart SE. Caveolin3 Stabilizes McT1-Mediated Lactate/Proton Transport in Cardiomyocytes. *Circ Res* 2021;**128**:E102–E120.

14. Voigt N, Trausch A, Knaut M, Matschke K, Varró A, Wagoner DR Van, Nattel S, Ravens U, Dobrev D. Left-to-right atrial inward rectifier potassium current gradients in patients with paroxysmal versus chronic atrial fibrillation. *Circ Arrhythmia Electrophysiol* 2010;**3**:472–480.

15. Voigt N, Friedrich A, Bock M, Wettwer E, Christ T, Knaut M, Strasser RH, Ravens U, Dobrev D. Differential phosphorylation-dependent regulation of constitutively active and muscarinic receptor-activated $I_{K,ACh}$ channels in patients with chronic atrial fibrillation. *Cardiovasc Res* 2007;**74**:426–437.

16. Dobrev D, Friedrich A, Voigt N, Jost N, Wettwer E, Christ T, Knaut M, Ravens U. The G protein-gated potassium current $I_{K,ACh}$ is constitutively active in patients with chronic atrial fibrillation. *Circulation* 2005;**112**:3697–3706.

17. Seibertz F, Rapedius M, Fakuade FE, Tomsits P, Liutkute A, Cyganek L, Becker N, Majumder R, Clauß S, Fertig N, Voigt N. A modern automated patch-clamp approach for high throughput electrophysiology recordings in native cardiomyocytes. *Commun Biol* 2022;**5**:969.

18. Jung P, Seibertz F, Fakuade FE, Ignatyeva N, Sampathkumar S, Ritter M, Li H, Mason FE, Ebert A, Voigt N. Increased cytosolic calcium buffering contributes to a cellular arrhythmogenic substrate in iPSC-cardiomyocytes from patients with dilated cardiomyopathy. *Basic Res Cardiol* 2022;**117**:5.

19. Wettwer E, Hála O, Christ T, Heubach JF, Dobrev D, Knaut M, Varró A, Ravens U. Role of $I_{Kur}$ in controlling action potential shape and contractility in the human atrium: Influence of chronic atrial fibrillation. *Circulation* 2004;**110**:2299–2306.

20. Kensah G, Gruh I, Viering J, Schumann H, Dahlmann J, Meyer H, Skvorc D, Bär A, Akhyari P, Heisterkamp A, Haverich A, Martin U. A novel miniaturized multimodal bioreactor for continuous in situ assessment of bioartificial cardiac tissue during stimulation and maturation. *Tissue Eng - Part C Methods* 2011;**17**:463–473.
